# Knockout of *ACTB* and *ACTG1* with CRISPR/Cas9(D10A) Technique Shows that Non-Muscle β and γ Actin Are Not Equal in Relation to Human Melanoma Cells’ Motility and Focal Adhesion Formation

**DOI:** 10.3390/ijms21082746

**Published:** 2020-04-15

**Authors:** Natalia Malek, Ewa Mrówczyńska, Aleksandra Michrowska, Ewa Mazurkiewicz, Iuliia Pavlyk, Antonina Joanna Mazur

**Affiliations:** Department of Cell Pathology, Faculty of Biotechnology, University of Wroclaw, 50-383 Wroclaw, Poland; en.malek@gmail.com (N.M.); ewa.mrowczynska@uwr.edu.pl (E.M.); 282709@uwr.edu.pl (A.M.); ewa.mazurkiewicz@uwr.edu.pl (E.M.); pavlykj@gmail.com (I.P.)

**Keywords:** beta actin, gamma actin, actin isoforms, melanoma, CRISPR/Cas9(D10A) technique, focal adhesion, adhesion, migration, LPA, PMA

## Abstract

Non-muscle actins have been studied for many decades; however, the reason for the existence of both isoforms is still unclear. Here we show, for the first time, a successful inactivation of the *ACTB* (CRISPR clones with inactivated *ACTB*, CR-*ACTB*) and *ACTG1* (CRISPR clones with inactivated *ACTG1*, CR-*ACTG1*) genes in human melanoma cells (A375) via the RNA-guided D10A mutated Cas9 nuclease gene editing [CRISPR/Cas9(D10A)] technique. This approach allowed us to evaluate how melanoma cell motility was impacted by the lack of either β actin coded by *ACTB* or γ actin coded by *ACTG1*. First, we observed different distributions of β and γ actin in the cells, and the absence of one actin isoform was compensated for via increased expression of the other isoform. Moreover, we noted that γ actin knockout had more severe consequences on cell migration and invasion than β actin knockout. Next, we observed that the formation rate of bundled stress fibers in CR-*ACTG1* cells was increased, but lamellipodial activity in these cells was impaired, compared to controls. Finally, we discovered that the formation rate of focal adhesions (FAs) and, subsequently, FA-dependent signaling were altered in both the CR-*ACTB* and CR-*ACTG1* clones; however, a more detrimental effect was observed for γ actin-deficient cells. Our research shows that both non-muscle actins play distinctive roles in melanoma cells’ FA formation and motility.

## 1. Introduction

The actin cytoskeleton is essential for the proper functioning of many cellular processes, including maintenance of the cell shape, chemotaxis, cell movement, adhesion, transport of cellular organelles, mitosis, replication, transcription, and even DNA repair [1,2]. Such a wide array of functions must be coordinated by several signaling pathways, which often intersect with each other, and a number of proteins. There are more than 200 actin-binding proteins (ABPs), which are responsible for the nucleation of actin polymerization as well as branching, crosslinking, and stabilization, but also severing of polymerized actin (filamentous actin, F-actin). Some ABPs, due to their activity in actin monomer sequestration, contribute to the maintenance of the cell’s monomeric actin pool (globular actin, G-actin) [3,4,5].

In a cell, there are linear and branched actin filament networks. The spatial organization of a particular actin network and the type of ABPs associated with that network determine the type of F-actin-based subcellular structure. The lamellipodium, which is involved in cell movement, is a dynamic structure that is composed of a dense network of short and branched actin filaments. Stress fibers, which consist of about 10–30 microfilaments aligned in parallel, are partly responsible for cell contraction and adhesion [3]. Other structures built by F-actin in tumor cells are invadopodia, which, due to the secretion of metalloproteases, are responsible for proteolytic degradation of the extracellular matrix and, thus, participate in the adhesion and migration/invasion of neoplastic cells [6].

In higher vertebrates, including mammals, there are four muscle isoforms and two non-muscle isoforms of actin [7,8,9,10], and although actins have been intensively studied for several decades, there are still controversies about the functional diversification of non-muscle isoactins (β and γ actin), which differ by only four amino acids within their N-termini [7,8]. Their different properties are explained, on the one hand, by protein features that manifest through dissimilar polymerization dynamics [11], divergent interactions with ABPs [7,12], and discrepancies in posttranslational modifications [13]. On the other hand, it is claimed that it is not the isoactins’ amino acid sequence but instead their nucleotide sequence that is more relevant for these differences [14], which are mirrored in mRNA trafficking [15] and the speed of isoactin biosynthesis [16]. However, the existence of two extremely similar actin isoforms has to be of high relevance since their expression patterns differ in various tissues (summarized in [7,8,10]), although their protein levels are similar in most cell types. Supporting this notion, mutations in genes encoding β and γ actins give different outcomes in, e.g., patients with Baraitser–Winter cerebrofrontofacial syndrome (BWCFF), where the cases with *ACTB* mutations are associated with severe forms of this condition, whereas cases with mutations in *ACTG1* are restricted to brain malformations [17,18]. 

Because there is no consensus regarding the purpose of the existence of two non-muscle actins, it is of high importance to study the functions of β and γ actin. Several published studies aimed to elucidate the roles of non-muscle actins by overexpression, silencing, or knockout of the coding sequences for β or γ actin (reviewed by [7]). However, in the overexpression and silencing studies, endogenous isoactins were present in the tested cells, which could have affected the results, whereas the knockout studies were conducted using normal cells, e.g., murine fibroblasts. In our studies, we focused on melanoma cells since this very aggressive tumor is characterized by its high plasticity [19] and the ability to form tumors from a single transformed cell [20]. This makes melanoma a perfect model to investigate the role of the aforementioned actin isoforms in migration. Moreover, as far as we know, there is no previous research that aimed to simultaneously estimate the role of the β and γ actins in melanoma cell migration. Here, by inactivation the genes encoding the β (*ACTB*) and γ (*ACTG1*) actins with the help of the RNA-guided D10A mutated Cas9 nuclease gene editing, CRISPR/Cas9(D10A), system in A375 cells, we report, for the first time, that the lack of γ actin had the most severe consequences on A375 cells and these non-muscle actins are unequal in their features, particularly their influence on the formation of focal adhesions (FAs) and protrusive structures and, thus, the motility of melanoma cells. 

## 2. Results

### 2.1. Both Non-Muscle Actins Are Highly Abundant in A375 Cells but Occupy Different Areas of the Cell

**Figure 1:**
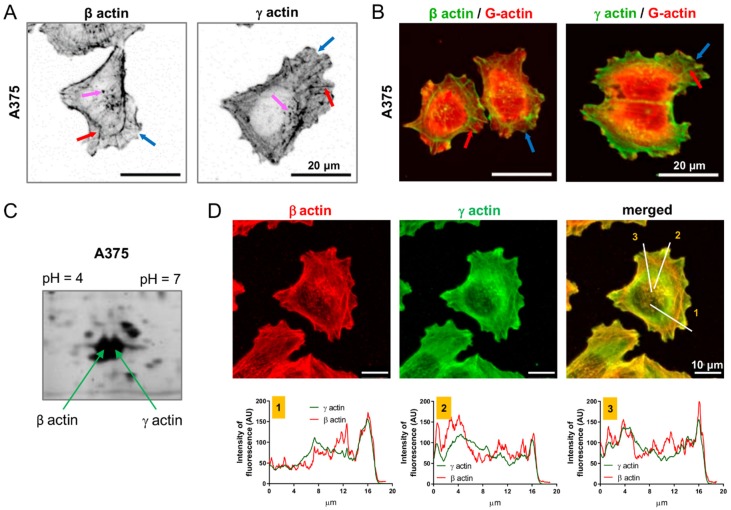
Differing distribution of non-muscle isoactins in A375 cells. (**A**) The cells were stained with antibodies detecting β and γ actin isoforms. Pink arrows point at invadopodia, red ones at stress fibers and blue ones at lamellipodia. (**B**) A375 cells growing on coverslips were fixed and stained to detect β or γ actin and globular actin by using appropriate antibodies and DNase I coupled to Alexa Fluor™ 594. Red arrows highlight stress fibers and blue ones lamellipodia. (**C**) Total protein extract from A375 cells (twenty μg of protein) was subjected to 2-D PAGE. The gel was stained with silver. (**D**) Immunocytochemically stained cells to detect β and γ actin. Three lines were drawn on a merged photo of the cell and the fluorescence histograms representing signal intensities for every fluorochrome were prepared on this basis.

Prior to subjecting the cells to the CRISPR/Cas9(D10A) procedure, we examined the expression level of both non-muscle actins in melanoma cells. In all the melanoma cell lines we tested, we noted various levels of the transcripts that encode both of the non-muscle isoactins (Figure S1). Upon normalization of the results to A375 cells there were statistically significant differences in *ACTB* and *ACTG1* expression for other melanoma cells lines when compared to A375 cells, however the differences were not so high varying form ca. 0.5- to 2-fold. Nevertheless, given the strict regulation of expression of the actin-encoding genes, even about twice as high *ACTB* expression (WM1341D cells) or twice as low *ACTG1* (WM9 cells) may have important implications for cell biology. For this study, we chose the A375 cell line as it is a cell line that was isolated from primary melanoma localized within skin with the potential to form metastases and it is easy to transfect. First, we looked at the distribution of β and γ actin in A375 cells. The specificity of the antibodies used in this study to detect both isoactins was checked using recombinant β and γ actin (Figure S2). Two types of antibodies against β actin and another two against γ actin were proven to be specific, as declared by the manufacturers. We noted that β actin in A375 was incorporated in F-actin structures, such as stress fibers and invadopodia, whereas γ actin, apart from being present in these structures as well, was also very prominently present as the dense actin mesh, both in the perinuclear area and within lamellipodia (Figure 1A). This was particularly easy to observe with double staining, where G-actin was detected together with β or γ actin (Figure 1B). Both actins seemed to be expressed at similar levels in these cells, as revealed by 2-D PAGE (Figure 1C). Simultaneous detection of both non-muscle actins in A375 cells revealed that the distribution of fluorescently labeled actins differed: β actin was more frequently located within actin bundles and γ actin at the periphery of the cell (Figure 1D). Histograms of the fluorescence signals that represent β and γ actin, conducted from the middle of the cell towards its border, clearly show that the fluorescence signal coming from the detection of β actin did not overlap with the γ actin fluorescence signal along the whole measured distance of the cell. However, intriguingly, a colocalization between these two non-muscle actins was observed at the cell cortex (Figure 1D). These observations indicate that both β and γ actin have partially different localizations in a cell. 

### 2.2. Successful Knockout of Genes Coding for β and γ Actin Leads to Altered Non-Muscle Isoactin Expression and Affects Cells’ Migration

**Figure 2:**
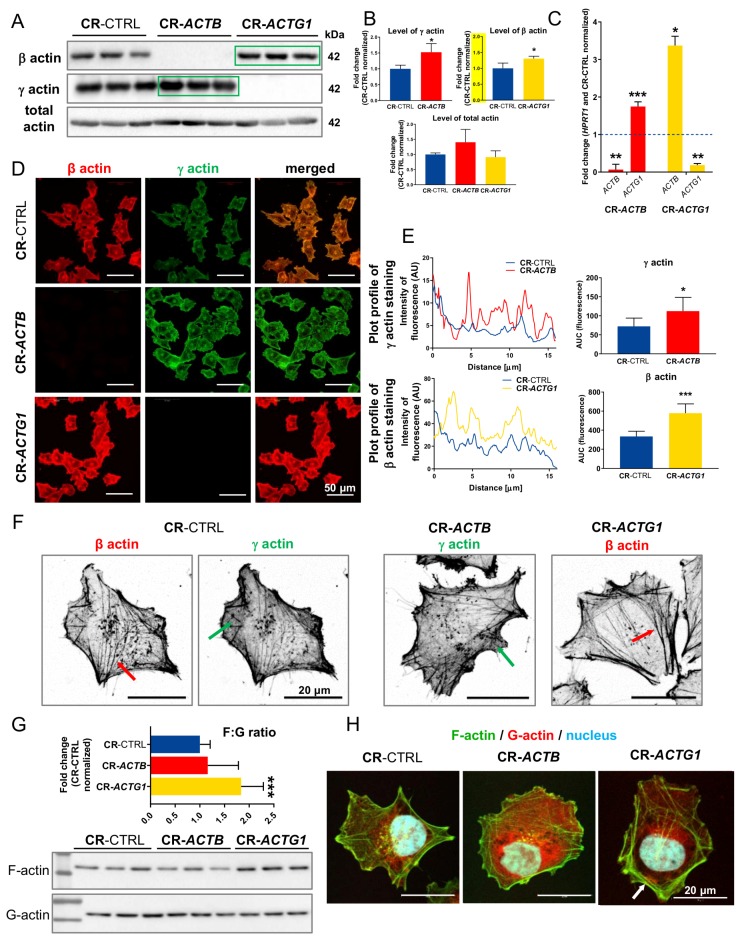
Successful inactivation of *ACTB* and *ACTG1* genes and altered actin cytoskeleton architecture in CR-*ACTB* [clones with inactivated *ACTB* with the help of CRISPR/Cas9(D10A) technique] and CR-*ACTG1* [clones with inactivated *ACTG1* with the help of CRISPR/Cas9(D10A) technique] clones. (**A**) Western blot analysis of control cells and clones without either β or γ actin. To identify isoactins and total actin, appropriate antibodies were used. Corresponding Ponceau S stainings of membranes are shown in Figure S6. Forty μg of protein was loaded on every lane. Green rectangles represent statistically higher level of protein in comparison to control cells. (**B**) Densitometric analysis of β, γ, and total actin level in tested clones done on membranes shown in (**A**) (*n* = 3). (**C**) The qRT-PCR analysis of *ACTB* and *ACTG1* expression level in CR-*ACTB* and CR-*ACTG1* clones (*n* = 9). (**D**) Clones devoid of β or γ actin were immunostained with antibodies recognizing non-muscle actins. All pictures were taken at the same settings used during microscopic observations. Pictures of two other clones for every condition are shown in Figure S4. (**E**) Representative plots presenting fluorescence distribution of stained isoactins in tested clones shown in (**D**). Quantitative analysis of area under curve (AUC) from plots (*n* = 6). (**F**) Clones were immunostained with anti-β and anti-γ actin antibodies to show cellular distribution of both isoactins. Red arrows point at stress fibers and green ones at actin mesh. (**G**) Western blot analysis of filamentous and globular actin levels in clones without β or γ actin growing on coverslips for 72 h. Nitrocellulose membranes were incubated with mouse anti-total actin antibodies. Quantitative estimation of filamentous (**F**) to globular (**G**) ratio (*n* = 8–9). (**H**) Clones were stained with fluorescently labelled phalloidin and DNase I to detect F- and G-actin. White arrows highlight F-actin accumulation. Results are expressed as the mean ±SD (**B**, **E**, **G**) or ±SEM (**C**), *p* ≤ 0.05 (*), *p* ≤ 0.01 (**), *p* ≤ 0.001 (***).

In order to test the hypothesis that β and γ actin have different functions in A375 cells, we decided to knock out the *ACTB* and *ACTG1* genes via the CRISPR/Cas9(D10A) technique. We successfully obtained these lines, which we confirmed by genomic DNA (gDNA) (Figure S3A), Western blot (Figure 2A,B and Figure S3B), and qPCR (Figure 2C) analyses and by immunocytochemical staining of the obtained clones (Figure 2D and Figure S4). Apart from cells devoid of β and γ actin synthesis, we also obtained control cells, i.e., clones transfected with CRISPR/Cas9(D10A) plasmids that code for scrambled guide RNAs, gRNAs. The control clones had normal levels of both of the non-muscle actins (Figure S3C). Interestingly, we observed compensation after the depletion of one isoform, i.e., upregulation of the level of the other isoform. This was seen in both the Western blot (Figure 2A,B) and microscopic analyses (Figure 2D,E, Figure S4). In the Western blot analysis, we observed this occurrence only if we lysed the cells in the lysis buffer used for 2-D PAGE, which contains high levels of urea and thiourea and, thus, better extracts the proteins associated with the detergent-resistant membrane fractions of the cells. When we used cytoskeletal-bound protein extraction buffer, we did not observe any compensation (Figure S5). For all the other Western blot analyses, we used cytoskeletal-bound protein extraction buffer. A closer look at the stained cells revealed (Figure 2F) that in the case of CR-*ACTB* [clones with inactivated *ACTB* with the help of CRISPR/Cas9(D10A) technique] cells, the elevated levels of γ actin manifested more prominently in structures that are characteristic for this isoform, as shown in Figure 1A,B. A similar observation was made for the β actin distribution in A375 CR-*ACTG1* [clones with inactivated *ACTG1* with the help of CRISPR/Cas9(D10A) technique] cells. Here, the stress fibers’ formation was more pronounced than in the control cells (Figure 2F); this was reflected in the elevated filamentous (F) to globular (G) actin ratio (Figure 2G) and more pronounced staining for F-actin (Figure 2H) in CR-*ACTG1* cells. In the case of the cells without β actin, the F:G actin ratio was unchanged in comparison to the control cells (Figure 2G) and the F-actin staining patterns were similar between the CR-*ACTB* clones and control cells (Figure 2H). These results show that the CRISPR/Cas9(D10A) technique is suitable for knocking out *ACTB* and *ACTG1* and that the obtained clones have altered actin cytoskeleton architecture.

### 2.3. Migration and Invasion Are Affected in the CR-ACTB Clones and Greatly Affected in the CR-ACTG1 Clones

**Figure 3:**
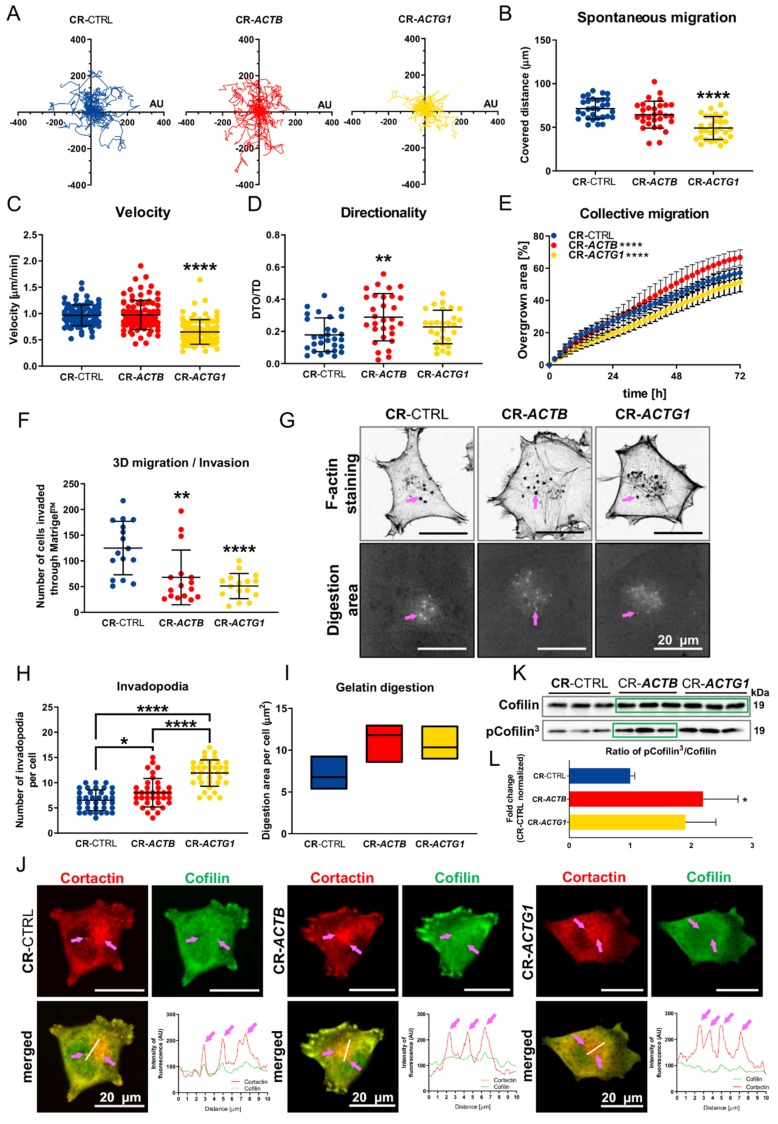
Cells devoid of β or γ actin had impaired invasion abilities, but only CR-*ACTG1* cells’ 2-D migration was affected. (**A**–**D**) Spontaneous 2-D migration of tested cells. The cells of studied clones growing on IncuCyte^®^ ImageLock plates were recorded over 72 h with IncuCyte^®^ Live Cell Analysis Imaging System (*n* = 30). (**A**) Trajectories of single cells’ migration. (**B**) Calculated covered distances of the cells. (**C**) Estimation of velocity and (**D**) directionality of cells’ migration. (**E**) Wound healing assay was performed on control (CR-CTRL), CR-*ACTB,* and CR-*ACTG1* cells to analyze collective migration over 72 h (*n* = 3). (**F**) Analysis of 3-D migration/invasion of control cells and cells without β or γ actin (*n* = 16). The cells were seeded onto Matrigel™ gel placed in the upper compartment of a Transwell™ and to the lower compartment a medium enriched in serum was added. (**G**) Gelatin digestion assay. The cells seeded on fluorescently labeled gelatin were 12 h later fixed and stained with Alexa Fluor™ 488 phalloidin. Bright spots represent digested gelatin by cells. Pink arrows point at invadopodia. (**H**) Quantitative analysis of invadopodia number and (**I**) dimensions of digested area (*n* = 36). (**J**) Cells after fixation were stained with antibodies recognizing cofilin and cortactin. Across the area rich in invadopodia a line was drawn and intensities of fluorescence for detected cortactin and cofilin were plotted on corresponding graphs. Pink arrows point at invadopodia. (**K**) Western blot analysis of cell lysates. Membranes were probed with antibodies detecting cofilin and cofilin phosphorylated at Ser3 (p-cofilin^3^). Corresponding densitometric analysis and Ponceau S stainings of membranes are shown in Figure S8. Forty μg of protein was loaded on every lane. Green rectangles represent statistically higher level of protein in comparison to control cells. (**L**) Calculated p-cofilin^3^:cofilin ratio (*n* = 3). Results are expressed as the mean ± SD (**B**–**F**,**H**,**K**) or median (**I**), *p* ≤ 0.05 (*), *p* ≤ 0.01 (**), *p* ≤ 0.0001 (****).

Because there were discrepancies, in terms of actin architecture, between the studied clones, we checked the influence of depletion of β or γ actin on the cells’ ability to migrate and invade. The distance covered by the CR-*ACTB* cells (Figure 3A,B) and their movement speed (Figure 3C) was similar to the results obtained for the control cells, but, surprisingly, the directionality of the β actin-devoid cells was changed (Figure 3D). In contrast, the 2-D migration of the CR-*ACTG1* clones was severely affected. The distances covered by these cells and their velocity were statistically significantly lowered in comparison to the control cells (Figure 3A–C), but their directionality was not (Figure 3D). The increased directionality of the CR-*ACTB* cells was reflected in the higher collective migration of these cells, compared to control cells (Figure 3E, Figure S7). Again, for the CR-*ACTG1* cells, we observed less migration than for the control (CR-CTRL) clones (Figure 3E, Figure S7). Next, we wanted to check the 3-D migration abilities of the studied cells and for that we used Transwell™ filters filled with Matrigel™ gel to mimic basement membrane. For the CR-*ACTB* and CR-*ACTG1* clones, we noted that fewer cells were capable of crossing the 3-D matrix towards the medium with 20% fetal bovine serum (FBS) (Figure 3F). Intriguingly, we observed that in these cells the number of invadopodia was higher than in the control cells (Figure 3G,H). This was even more drastic in the case of CR-*ACTG1* cells since the number of invadopodia was much higher than in the CR-*ACTB* clones. Additionally, we observed that the area digested by metalloproteases in the CR-*ACTB* and CR-*ACTG1* cells was larger than that of the control cells, although the differences were not statistically significant (Figure 3I). Next, we stained the cells for cofilin and cortactin, two proteins that are important for invadopodia formation [21,22]. This staining revealed that both cofilin and cortactin are indeed present in these structures (Figure 3J); however, cofilin, unlike cortactin, is not present to the same extent in invadopodia for the CR-*ACTB* and CR-*ACTG1* cells as it is in the control cells. This was especially evident in the fluorescence intensity plot profiles of the presence of cortactin and cofilin at invadopodia (Figure 3J). Afterwards, we checked the levels of cofilin and its phosphorylated form (p-cofilin). We noted that the levels of total cofilin were highly increased in the CR-*ACTB* and CR-*ACTG1* cells, when compared to control clones (Figure 3K, Figure S8); however, we only observed a statistically higher amount of p-cofilin^3^ in the CR-*ACTB* cells, compared to the control clones (Figure 3K, Figure S8). We estimated the p-cofilin^3^:cofilin ratios for all the cell types and noted that the ratio statistically significantly increased only for CR-*ACTB* cells, although we observed a similar tendency in the CR-*ACTG1* cells (Figure 3L). In summary, a lack of γ actin has more severe consequences on the cells’ 2-D and 3-D migration than a lack of β actin. 

### 2.4. Bundling of Stress Fibers Is Altered in Clones that Lack Either β or γ Actin

**Figure 4:**
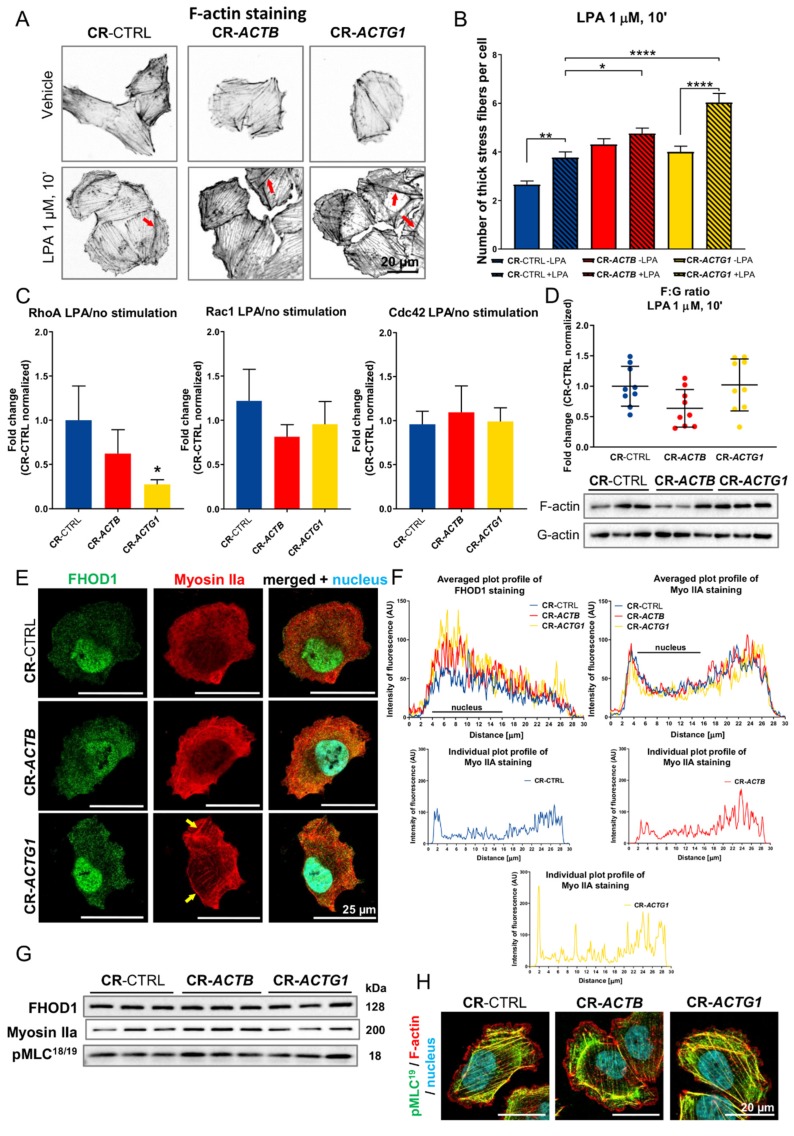
Stress fibers’ formation is disturbed in clones devoid of β or γ actin. (**A**) CR-CTRL, CR-*ACTB,* and CR-*ACTG1* cells upon stimulation with LPA were fixed and stained with Alexa Fluor™ 488 phalloidin. Red arrows point at stress fibers. (**B**) The number of thick stress fibers was calculated for every condition and presented as a bar chart (*n* = 61–81). (**C**) Activity of RhoA, Rac1, and Cdc42 was estimated after lysophosphatidic acid (LPA) stimulation (*n* = 3). (**D**) F:G actin ratio was evaluated in the cells on the base of Western blot analysis of F- and G-actin levels in studied clones (*n* = 9). Membranes were incubated with anti-total actin antibodies. (**E**) The cells were immunostained with antibodies detecting the human FH1/FH2 domain-containing protein 1 (FHOD1) and Myosin IIa. Yellow arrows highlight Myosin IIa accumulation. (**F**) Plots of averaged (*n* = 9) and individual fluorescence intensity distribution for FHOD1 and Myosin IIa staining across a cell. (**G**) Western blot analysis of cell lysates. Membranes were probed for FHOD1, Myosin IIa, and myosin light chain phosphorylated at Thr18 and Ser19 (pMLC^18/19^). Corresponding densitometric analysis and Ponceau S stainings of membranes are shown in Figure S9. Forty μg of protein was loaded on every lane. (**H**) The cells were stained with anti-pMLC^19^ antibodies and fluorescently labeled phalloidin. Results are expressed as the mean ± SD (**C**,**D**) or ± SEM (**B**), *p* ≤ 0.05 (*), *p* ≤ 0.01 (**), *p* ≤ 0.0001 (****).

Subsequently, we checked the formation of F-actin-rich structures as they are directly involved in cell motility. We incubated cells that were starved for 24 h with 1 micromolar (μM) lysophosphatidic acid (LPA) for 10 min in order to challenge the cells to form stress fibers [23]. This was achieved (Figure 4A), and we calculated the number of “thick” stress fiber bundles, plotting a histogram of fluorescence over the cell length and identifying the number of fluorescent values that were higher or equal to 75% of the highest fluorescence peak. The highest number of these structures was noted in the CR-*ACTG1* clones with LPA stimulation (Figure 4B). Interestingly, in the case of control cells and CR-*ACTG1* cells, there were more thick stress fiber bundles after stimulation than in nonstimulated cells, whereas this was not true in the CR-*ACTB* cells. We then looked at activation of Rho-GTPases upon LPA stimulation, as LPA is a Rho activator [23]. After the addition of LPA, a statistically significant decrease in the level of active RhoA was observed only in the CR-*ACTG1* cells (Figure 4C). The levels of Rac1 and Cdc42 activity were similar between the clones (Figure 4C). Next, we evaluated the F:G actin ratio; this ratio was similar in all three cell types (Figure 4D), although there was a tendency toward a decrease for the CR-*ACTB* cells. Next, we looked at the distribution of the human FH1/FH2 domain-containing protein 1, FHOD1, formin (a microfilament bundling and capping protein [24]), and Myosin IIa, an actin motor protein that possesses microfilament bundling activity [25]. While we did not observe any changes in the cellular distribution of FHOD1, the Myosin IIa staining pattern was altered in CR-*ACTG1* cells (Figure 4E). The signal for Myosin IIa in these cells was not homogenously distributed, as in the control cells, but instead clear, thick bundles were seen. Although this was not reflected in the results of the analysis of averaged histograms, which represent the fluorescence signal of detected FHOD1 or Myosin IIa, it was mirrored in the plot profiles of representative cells for every condition (Figure 4F). Moreover, the FHOD1 and Myosin IIa levels were similar in the CR-*ACTB*, CR-*ACTG1*, and control cells (Figure 4G, Figure S9). We also checked the phosphorylated myosin light chain (pMLC) levels, as pMLC is an indicator of myosin ATPase activity and, thus, activation of the contractile apparatus [26]. The pMLC levels were similar among all the cell types (Figure 4G, Figure S9). Although there were no differences in the levels of Myosin IIa or pMLC between the different clones after LPA stimulation, when grown in full medium, significantly higher levels of Myosin IIa and pMLC were exhibited by the CR-*ACTB* cells and the CR-*ACTG1* clones had lower basal levels of FHOD1 (Figure S10). Finally, we stained the cells for pMLC and we noticed a strong decoration of stress fibers with pMLC in control and CR-*ACTG1* cells, but not in the CR-*ACTB* clones (Figure 4H). These observations imply that the formation rate of stress fibers is more enhanced in the cells devoid of γ actin than in control cells or cells without β actin. 

### 2.5. Lamellipodial Dynamics Are Altered in Cells Devoid of Either β or γ Actin

**Figure 5:**
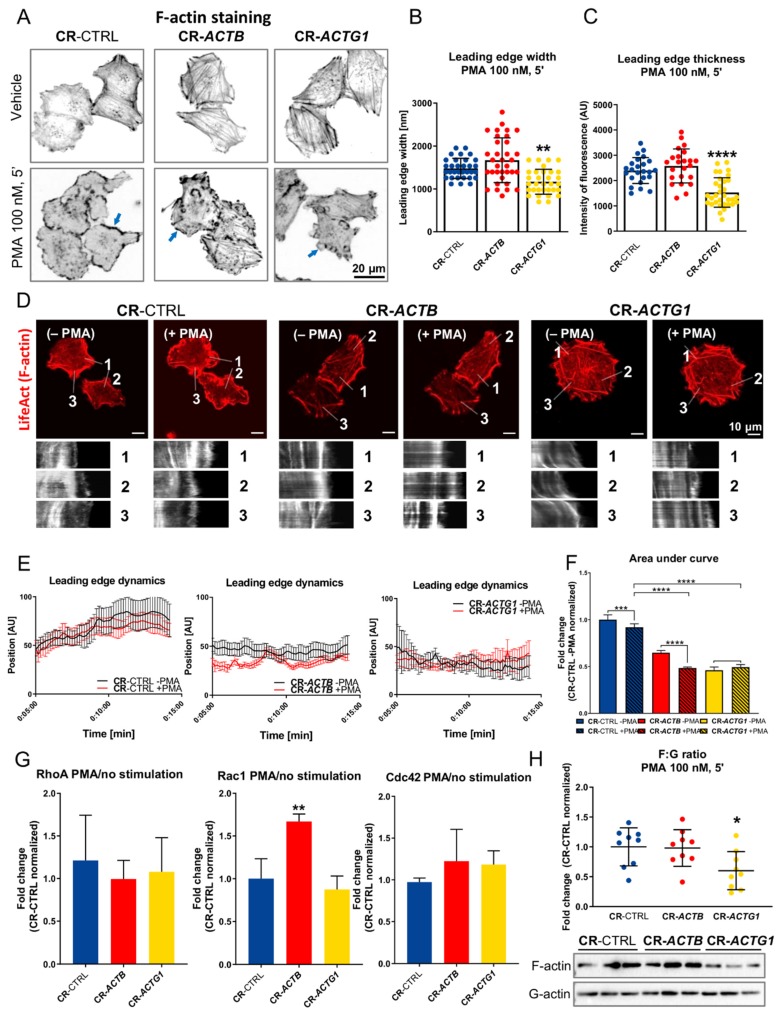
Lamellipodial dynamics are altered in cells devoid of β or γ actin. (**A**) Control clones and clones devoid β or γ actin after 5 min of incubation with phorbol-12-myristate-13-acetate (PMA) were fixed and F-actin was stained with the help of Alexa Fluor™ 488 phalloidin. (**B**) Quantification of leading edge width upon incubation with PMA (*n* = 32–34). (**C**) Estimation of leading edge thickness of cells exposed to PMA (*n* = 23–32). (**D**) Representative movie frame with marked lines from which kymographs were created. (**E**) Plots showing positon of the leading edge represented by F-actin detection under control and PMA exposition conditions (*n* = 6). (**F**) Calculated AUC from plots presented in (**E**). (**G**) Estimation of activity of RhoA, Rac1, and Cdc42 was upon PMA stimulation (*n* = 3). (**H**) F:G actin ratio estimation (*n* = 9). The membranes were probed for total actin. Blue arrows point at lamellipodia. Results are expressed as the mean ± SD (**B**,**C**,**F**–**H**) or ± SEM (**E**), *p* ≤ 0.05 (*), *p* ≤ 0.01 (**), *p* ≤ 0.001 (***), *p* ≤ 0.0001 (****).

Since we checked the clones’ ability to form stress fibers, we also looked at the other F-actin protrusive structures, lamellipodia. Phorbol-12-myristate-13-acetate (PMA) stimulation was performed to induce formation of lamellipodia [27]. F-actin staining in fixed cells and analysis of micrographs revealed that cells devoid of γ actin formed narrower and thinner leading edges than did control cells (Figure 5A–C). The width and the thickness of lamellipodium were measured by plotting a histogram of fluorescence over the lamellipodium, and using a ruler in the software or reading the whole intensity of fluorescence through the lamellipodium, respectively. In the case of the CR-*ACTB* cells, there were no differences in these aspects compared to controls. Afterwards, we performed live imaging analysis of clones that were transfected with a plasmid that encoded LifeAct-TagRFP in order to track F-actin in real time upon PMA stimulation. Analysis of the kymographs (Figure 5D) showed that the dynamics of F-actin within the leading edge were more prominent in CR-*ACTB* cells than in control cells (Figure 5D–F). Although, in the CR-*ACTG1* cells, there was no difference in the lamellipodial dynamics between the PMA-stimulated and nonstimulated groups, the overall lamellipodial dynamics were lower in those cells in comparison to those present in the controls (Figure 5F). Next, we evaluated the activation of Rho-GTPases upon PMA stimulation versus nonstimulated cells. We evaluated activity of GTPases with activation assays (G-LISA^®^) detecting given GTPase bound with GTP in cells lysates. Increased Rac1 activity was noted in CR-*ACTB* cells (Figure 5G); however, we did not observe any changes in the RhoA or Cdc42 activity between the clones in response to PMA treatment. The F:G actin ratio was also checked and a statistically significant decrease was noted only for the CR-*ACTG1* cells (Figure 5H). Altogether, these observations imply that while the leading edge dynamics were increased in CR-*ACTB* cells, the opposite effect was observed for CR-*ACTG1* cells, when compared to control cells. 

### 2.6. There Are Differences in the Distribution and Levels of Actin Polymerization Nucleators within the Leading Edge

**Figure 6:**
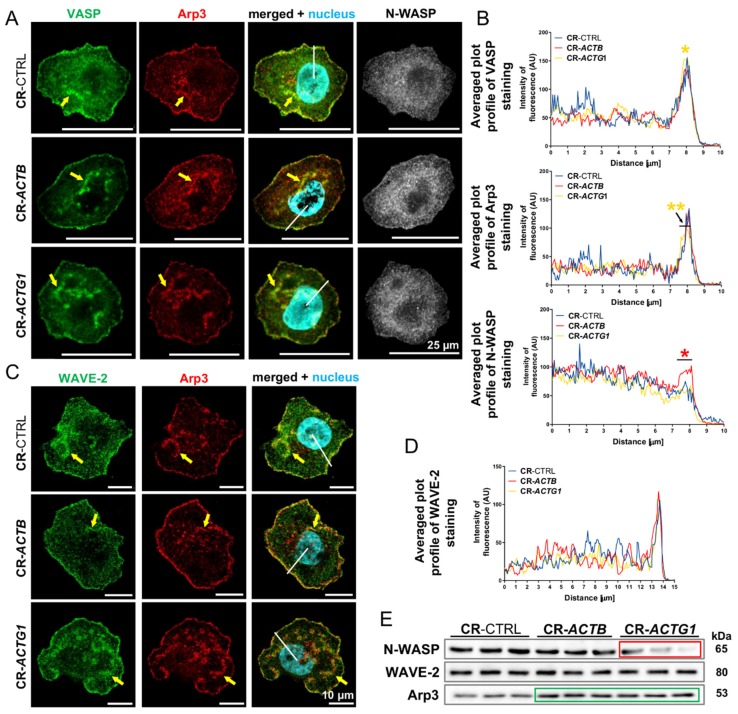
Changed distribution of actin binding proteins (ABPs) within leading edge upon lamellipodium formation challenge. (**A**) The cells were stained with vasodilator-stimulated phosphoprotein (VASP), actin-related protein 3 (Arp3), and neural Wiskott-Aldrich syndrome protein (N-WASP), recognizing antibodies. Yellow arrows point at circular dorsal ruffles. (**B**) Plots of averaged fluorescence intensity distribution of stained VASP, Arp3, and N-WASP. Lines were drawn from pericentral region of a cell (position 0 μm) towards the border of a cell (10 μm) (*n* = 9). Representative white lines are shown on merged microphotographs in (**A**). Black arrow points at the range for which there is a statistically significant difference for CR-*ACTG1* cells in comparison to control cells. (**C**) The cells were stained using specific antibodies to detect Wiskott-Aldrich syndrome protein family member 2 (WAVE-2) and Arp3. Yellow arrows point at circular dorsal ruffles. (**D**) Plots of averaged fluorescence intensity distribution for WAVE-2 from the center of a cell (position 0 μm) towards the border of a cell (position 15 μm). Representative white lines are shown on merged microphotographs in (**C**). (**E**) Cell lysates were analyzed by Western blotting. Membranes were incubated with antibodies directed against N-WASP, WAVE-2, and Arp3. Corresponding densitometric analysis and Ponceau S stainings of membranes are shown in Figure S11. Forty μg of protein was loaded on every lane. Green and red rectangles represent statistically higher and lower levels of protein, respectively, in comparison to control cells. Results are expressed as the mean only, *p* ≤ 0.05 (*), *p* ≤ 0.01 (**).

Because we noticed divergences between the clones in terms of their leading edge dynamics, we decided to look at proteins that trigger actin polymerization and reside in the leading edge. Upon exposure to PMA, the cells were fixed and stained for vasodilator-stimulated phosphoprotein (VASP) and actin-related protein 3 (Arp3) as these proteins are the nucleators of actin polymerization within the lamellipodium [28]. We found that both proteins were prominent at the leading edges of all the analyzed cell types; however, the presence of VASP was more pronounced in the CR-*ACTG1* clones, whereas the Arp3 level was diminished in these cells (Figure 6A,B). The presence of neural Wiskott-Aldrich syndrome protein (N-WASP) and Wiskott-Aldrich syndrome protein family member 2 (WAVE-2), two activators of Arp3, was also evaluated; while the amount of N-WASP was significantly increased at the leading edges of CR-*ACTB* cells, there were no observed differences in the amount of WAVE-2 between the tested clones (Figure 6C,D). Interestingly, we observed that VASP colocalized with Arp3 within circular dorsal ruffles (CDRs) in all the tested clones (Figure 6A). We also noticed the presence of Arp3 and WAVE-2 at the leading edges and CDRs in all the tested clones (Figure 6C). Western blot analysis showed that there was less N-WASP in the CR-*ACTG1* cells, whereas the level of Arp3 was increased in the CR-*ACTB* and CR-*ACTG1* clones, compared to controls (Figure 6E, Figure S11). We also checked the levels of those proteins under full medium conditions. The level of N-WASP was significantly statistically higher in both the CR-*ACTB* and CR-*ACTG1* cells when compared to control clones (Figure S12). Similarly, there was an increase in the level of Arp3 in both CRISPR clone types when compared to controls. There were no notable changes in the levels of WAVE-2 between the cell types (Figure S12). These observations suggest that alterations in the leading edge dynamics could be at least partially caused by changes in the subcellular distribution and amount of the proteins studied here. 

### 2.7. Focal Adhesion (FA) Formation Is Changed in the Clones Devoid of Either β or γ Actin

**Figure 7:**
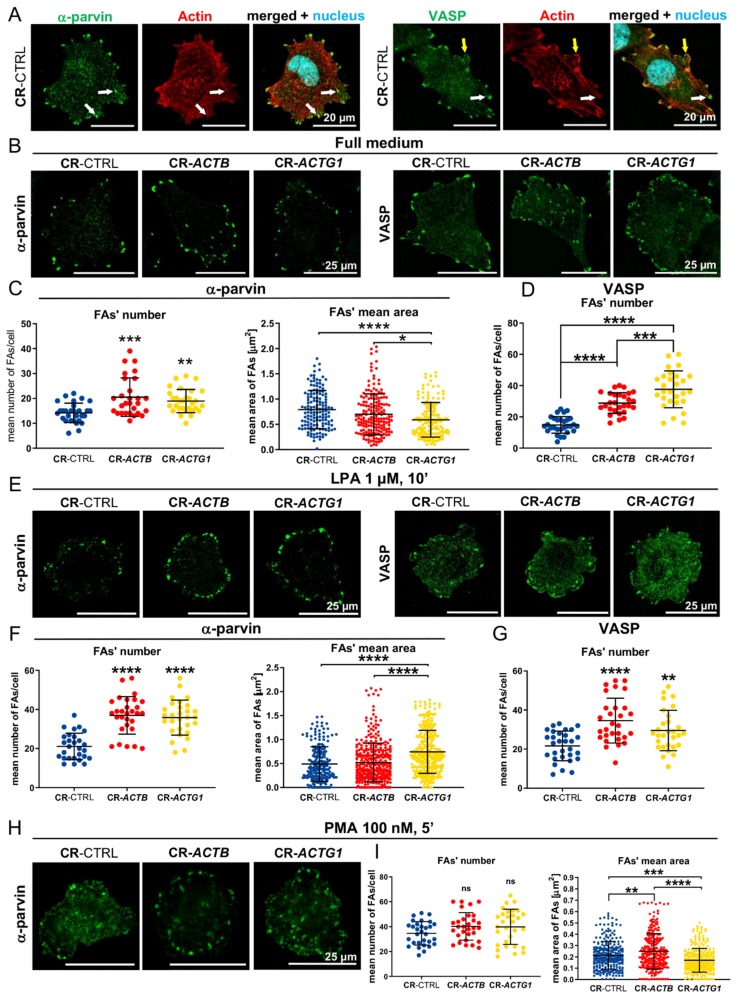
Focal adhesion formation is altered in clones devoid of β or γ actin. (**A**) Immunostaining detecting α-parvin, VASP, and total actin. The genetically nonmanipulated A375 cells were grown under full medium conditions. White arrows point at focal adhesions, while yellows ones at nascent focal adhesions (FAs)/accumulation of VASP. (**B**,**E**,**H**) The clones growing either in full medium or incubated with LPA or PMA were fixed and stained with antibodies recognizing α-parvin or VASP. (**C**,**F**,**I**) The number of α-parvin-rich focal adhesions as well as their surface area were calculated for every tested condition (*n* = 30). (**D**,**G**) The number of VASP-rich focal adhesions was calculated (*n* = 30). Results are expressed as the mean ± SD, *p* ≤ 0.05 (*), *p* ≤ 0.01 (**), *p* ≤ 0.001 (***), *p* ≤ 0.0001 (****).

Because VASP is not only found at the leading edge of a cell, but also in FAs and we have noticed differences in its presence at the leading edge between cell types, we decided to evaluate the FA formation in cells grown in full medium or under LPA or PMA stimulation conditions. For this purpose, we stimulated the cells, fixed them, and stained for two proteins present in FAs, VASP and α-parvin. Both proteins colocalized with integrin αVβ3, a known constituent of FAs (Figure S13). Closer analysis of stained cells revealed that VASP is also present in these FAs, just like α-parvin, but not only there; there is a subtle accumulation of VASP in the submembranous area of a cell, which is highlighted by arrows in Figure S13. The same was observed in nonmanipulated A375 cells stained with antibodies that recognize VASP or α-parvin and total actin (Figure 7A). In the case of α-parvin, we observed this protein solely at the tips of stress fibers. On the other hand, we noted a clear VASP localization at the tips of stress fibers, but also in submembranous aggregates that were also positive for total actin (Figure 7A). We consider these structures as nascent FAs that were most likely devoid of α-parvin at this stage of formation. Using photos of the cells with immunofluorescently detected α-parvin and VASP, we calculated and measured the number of FAs and their areas, respectively. In the case of staining with anti-VASP antibodies, some puncta were so small that we did not include measurements of their areas in order to avoid introducing any artifacts, and, therefore, the FAs’ areas were measured only for α-parvin-positive FA staining. The acquired results show that, under full medium conditions, the number of α-parvin-rich FAs was statistically significantly elevated in CR-*ACTB* and CR-*ACTG1* cells, in comparison to control cells, but, surprisingly, their surface area was decreased only in the CR-*ACTG1* clones (Figure 7B,C). There were more VASP puncta in cells without β actin when compared to the control clones, whereas the greatest number of VASP puncta was observed in the CR-*ACTG1* cells (Figure 7B,D). LPA incubation of the CR-*ACTB* and CR-*ACTG1* cells resulted in almost twice as many α-parvin-rich FAs in comparison to controls, whereas their surface area was increased only in the cells devoid of γ actin (Figure 7E,F). The number of VASP-rich nascent FAs and FAs was increased in both the β and γ actin knockout cells upon LPA stimulation (Figure 7E,G). We also checked the impact of PMA on the number of FAs. We noted no changes in the number of α-parvin-rich FAs in CR-*ACTB* and CR-*ACTG1* cells in comparison to controls (Figure 7H,I). The FA surface area was bigger in the clones without β actin, but surprisingly smaller in the CR-*ACTG1* cells, compared to control cells. 

**Figure 8:**
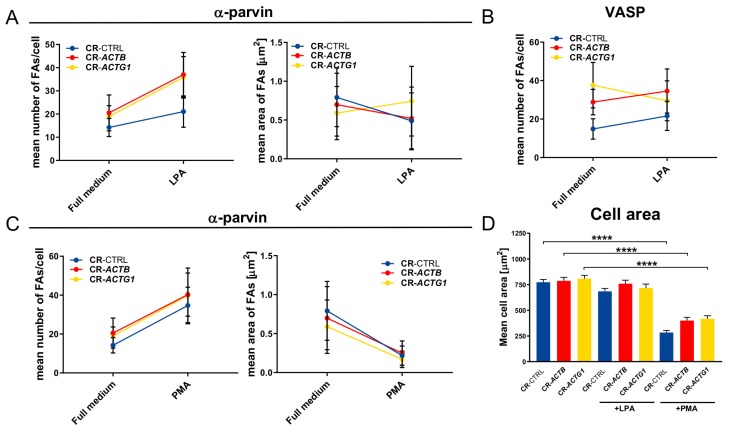
Trends in FAs’ formation by the studied clones and their surface area. (**A**–**C**) Slope graphs presenting trends upon LPA or PMA stimulation in number and surface area of focal adhesion detected either by staining with α-parvin or VASP-detecting antibodies (*n* = 30). (**D**) Clones’ cells surface area upon LPA and PMA stimulation was calculated and presented as a bar chart (*n* = 30). Results are expressed as the mean ± SD (**A**–**C**) or ± SEM (**D**), *p* ≤ 0.0001 (****).

Subsequently, we compared the results for all the clones concerning the numbers and sizes of FAs upon LPA or PMA stimulation under full medium conditions by employing slope graphs. We observed the same tendency for all the tested cells, i.e., LPA and PMA incubation increased the number of α-parvin-rich FAs and PMA treatment decreased FA size (Figure 8A,C). In the case of CR-*ACTG1* cells, we noted, surprisingly, an increase in the size of α-parvin-rich FAs upon LPA stimulation when compared to controls and CR-*ACTB* cells, for which a decrease was noted (Figure 8A). Similarly, after LPA treatment, the number of VASP-rich FAs decreased in the cells devoid of γ actin and increased in the control and CR-*ACTB* cells (Figure 8B). These results indicate that FA formation differed between the clones and that CR-*ACTG1* cells had impaired FA turnover. 

Finally, we evaluated the surface area of the cells under all the tested conditions. Although there were discrepancies in the numbers and sizes of FAs between the studied clones, the cell surface areas were similar between these clones under all the tested conditions (Figure 8D). However, upon PMA stimulation, the average cell size decreased in comparison to the no-treatment and LPA conditions. 

### 2.8. Signaling Connected to FAs Is Altered in the Clones Devoid of Either β or γ Actin

**Figure 9:**
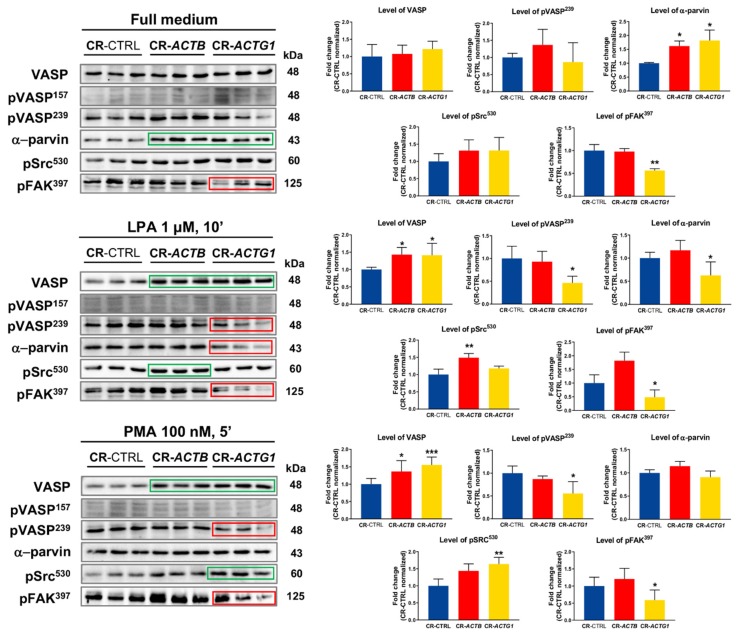
Changed FAs’ signaling in CR-CTRL, CR-*ACTB,* and CR-*ACTG1* clones. Western blot analysis of control clones and clones without given isoactin. Corresponding Ponceau S stainings of membranes are shown in Figure S14. Forty μg of protein was loaded on every lane. Membranes were probed for proteins found within focal adhesions and playing a significant role in FAs’ signaling: VASP, phosphorylated at Ser157 VASP (pVASP^157^), phosphorylated at Ser239 VASP (pVASP^239^), α-parvin, phosphorylated at Tyr530 proto-oncogene tyrosine-protein kinase Src (pSrc^530^), and phosphorylated at Tyr397 focal adhesion kinase 1 (pFAK^397^). Densitometric analysis of studied proteins levels in tested clones was done and presented as bar charts (*n* = 3–6). Green and red rectangles represent statistically higher and lower level of protein, respectively, in comparison to control cells. Results are expressed as the mean ± SD, *p* ≤ 0.05 (*), *p* ≤ 0.01 (**), *p* ≤ 0.001 (***).

Consequently, to follow on the observed changes in FA formation, we decided to check the levels of some FA structural proteins that play significant roles in FA-associated signaling, using Western blotting. We estimated the levels of VASP, phosphorylated VASP (pVASP^157^ and pVASP^239^, VASP forms without actin-binding activity [29]), α-parvin, and the phosphorylated forms of proto-oncogene tyrosine-protein kinase Src (Src) (pSrc^530^, inactive form of Src [30]) and focal adhesion kinase 1 (FAK) (pFAK^397^, active form of FAK [31]). In CR-*ACTB* and CR-*ACTG1* cells, we observed elevated level of VASP under full medium, LPA, and PMA conditions (Figure 9). Although we did not observe any signal for pVASP^157^, we noted a lower level of pVASP^239^ in the cells devoid of γ actin that were grown under LPA and PMA conditions (Figure 9). Interestingly, the α-parvin amount increased in the CR-*ACTB* and CR-*ACTG1* clones that grew in full medium, but decreased in the cells without γ actin upon exposure to LPA. LPA incubation upregulated the pSrc^530^ level in β actin-deficient clones, but PMA stimulation upregulated pSrc^530^ in the γ actin-deficient clones. Next, we looked at FAK activation and we noticed a decrease in the pFAK^397^ level in CR-*ACTG1* cells under all the tested conditions, compared to control cells. In summary, these results clearly show that altered FA formation in CR-*ACTB* and CR-*ACTG1* clones has a direct impact on FA-associated signaling.

## 3. Discussion

There is a constantly expanding plethora of questions to be answered regarding actin isoforms’ diversification to fully understand their involvement in spatiotemporal actin cytoskeleton regulation, which is crucial for the vast majority of cellular processes [8]. In regard to melanoma cells, there is only one paper that addresses non-muscle actins’ role in these cells. That study showed, based solely on qRT-PCR analysis, that there was increased β actin expression in invasive T1C1 cells in comparison to non-invasive 1C8 cells [32]. Because there was a gap in the knowledge about non-muscle actins’ involvement in melanoma cell biology, we decided to unveil the role of both isoactins in melanoma cells’ motility. 

We noted that in genetically nonmanipulated A375 cells, β actin is predominantly present in stress fibers, whereas γ actin is found mainly as a dense mesh in the cell body but also in stress fibers. Similar non-muscle isoactin subcellular localization was also observed by Dugina and her colleagues in fibroblasts, where β actin was predominantly present at cell–cell contact sites, in stress fibers, and in circular bundles, whereas γ actin was observed as a meshwork at the cortex and lamellipodium [33]. For colon carcinoma, lung adenocarcinoma cells, and keratinocytes, Dugina et al. obtained similar results, showing that β actin is mainly found in stress fibers, whereas γ actin is localized submembranously [34]. Analogous results were obtained by another group for neuroblastoma cells [35]. On the other hand, several papers have reported β actin localization in the lamellipodium [36,37,38] and γ actin in microfilaments in the cytoplasm [15,37]. Several studies reported compensation for the loss of one non-muscle actin isoform with overexpression of another actin isoform to restore the level of total actin [39,40,41]; we observed the same phenomenon. Higher expression of β or γ actin in the CR-*ACTG1* or CR-*ACTB* clones, respectively, was corroborated by microscopic analysis. 

Intriguingly, we found out that knocking out either β or γ actin influenced the expression levels of some ABPs. The expression of several ABPs is regulated by serum response factor (SRF) [42]; however, our observations cannot be solely explained by changes in the F:G ratio leading to altered SRF signaling, resulting in up- or down-regulation of ABP expression. We justify our notion by the fact that VASP, α-parvin, and N-WASP were up- or down-regulated in CR-*ACTB* and CR-*ACTG1* cells under various conditions, whereas the expression of these proteins is not regulated by SRF. On the other hand, we noted changed levels of cofilin and Arp3, proteins whose expression depends on SRF [42], in the cells deprived of either β or γ actin. However, in CR-*ACTB* clones, there were no changes in the F:G actin ratio. Alterations in ABPs expression levels were also noted by Latham and colleagues, who defined *ACTB*-AST, a new clinical entity manifested by microcephaly, intellectual disability, minor facial anomalies, white blood cell anomalies and thrombocytopenia caused by mutations clustered in the 3′ region of *ACTB* [39]. They noted the significantly increased expression of some ABPs: The α-actinin, myosin 2A, filamin A, and tropomyosin 4.2. Except for tropomyosin 4.2, the other proteins were recruited at a higher extent to F-actin bundles built by the mutant β actin and α-smooth muscle actin (α-SMA) in patient-derived fibroblasts, compared to control cells. Other research showed that mouse myoblasts that overexpressed β actin also exhibited elevated levels of thymosin β and actin-depolymerizing factor (ADF) [43]. Recent findings show that the cell’s different actin networks have to compete for the same restricted pool of G-actin [44,45,46]. The new, exquisite work from Antkowiak and his colleagues provides more details into this picture [47]. They showed that Arp2/3, profilin, Cap1/2, ADF/cofilin, and tropomyosin and their varying concentrations differently affected the balance between linear, formin-based, and Arp2/3 branched networks in both in vitro and in vivo models. The notion that the changes in the composition and dynamics of particular actin networks globally influence the pool of monomeric actin, and thus all F-actin structures present in the cell, was recently called “global treadmilling” by Carlier and Shekhar [28]. In light of the data presented here, it seems interesting that in the CR-*ACTG1* cells, the lamellipodial activity and Arp3 presence at the leading edge were diminished, while the formation of thick stress fiber bundles was prominently enhanced when compared to control and CR-*ACTB* cells. These observations are consistent with other studies that show that reduced activity of the Arp2/3 complex resulted in a disturbance in the formation of branched networks in favor of more extensive linear networks [48,49,50]. 

There are studies showing that β and γ actin have different affinities in terms of interaction with ABPs. For instance Müller with colleagues [51] proved that β actin more strongly activates non-muscle myosin 2C1, while γ actin preferentially activates myosin 7A. Dugina et al. [34], on the other hand, by application of co-immunoprecipitation and proximity ligation assay, showed that γ and not β actin associates with cofilin 1, PP1, WAVE2, and p34-Arc in lung adenocarcinoma A549 cells. Thus it is plausible to expect that the action of ABPs on actin cytoskeleton could be dependent on β:γ actin ratio. The issue of the capability of different isoactin monomers to copolymerize has been discussed for many years. Recently, it was shown by Bergeron and colleagues [11] that β and γ actin can copolymerize. Different β:γ actin ratio in microfilament composition could determine the filament polymerization dynamics and ABPs’ interaction kinetics and finally availability of polymerizable pool of actin monomer. In consequence, this could be mirrored in the character and dynamics of F-actin structures and finally would determine the cell behavior. The observed in this study effects of *ACTB* and *ACTG1* knock outs might be caused by either the lack of given actin isoform or the up-regulated level of the other one or by both. 

**Figure 10:**
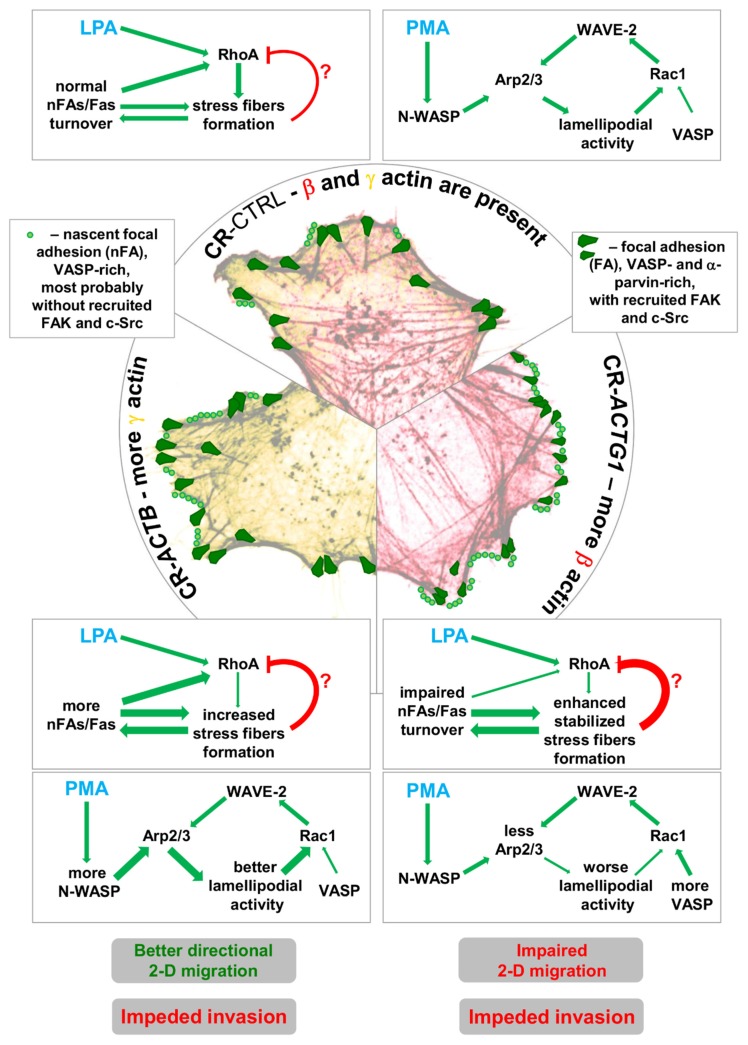
Proposed mechanism of influence of disturbed actin organization in CR-*ACTB* and CR-*ACTG1* cells on melanoma cells’ motility and adhesion. Cells devoid of β actin that grew in full medium exhibited more nascent (VASP-rich) focal adhesions (nFAs) and mature (VASP- and α-parvin-rich) focal adhesions (FAs) than did CR-CTRL cells. Although the CR-*ACTG1* and CR-*ACTB* cells possess more mature FAs under the same conditions than control cells, FAs of CR-*ACTG1* cells are smaller. Moreover, CR-*ACTG1* cells have many more nascent FAs than both control and CR-*ACTB* cells. Alterations in FA dynamics, stress fiber formation, and the distribution of some ABPs at the leading edge results in changes in Rac1 activation, improved 2-D migration, and impaired invasion in CR-*ACTB* cells. In the case of CR-*ACTG1* cells, the impaired FA turnover, increased number of thick stress fibers, and decreased level of Arp3 at the leading edge led to lowered activation status of RhoA and finally to impeded 2-D and 3-D migration. For details, please see the discussion. In summary, the lack of γ actin has more severe effects on melanoma cells than the lack of β actin. We believe that changes in the formation/turnover of FAs might be caused by the different properties of non-muscle actins’ interactions with ABPs.

The activity of Rho-GTPases regulates the formation of F-actin-based structures (lamellipodia, filopodia, and stress fibers) and thus migration [52]. Here, however, we do not see a clear correlation between Rho-GTPase activity upon LPA and PMA treatment and the F:G ratio or the formation of lamellipodia and stress fibers in the tested clones. We believe that the observed alterations in Rac1 and RhoA activity are a downstream effect of the altered actin network dynamics in the clones devoid of β or γ actin due to two possible mechanisms (Figure 10). In the case of CR-*ACTB* cells challenged with PMA, there was more N-WASP at the leading edges of the cells, which allowed more Arp2/3 to be activated and this resulted in better lamellipodial activity. As a result, this could eventually lead to higher Rac1 activity in comparison to control and CR-*ACTG1* cells since, as discussed by Dang and Gautreau, lamellipodial activity might have a positive feedback loop on Rac1 activity [53]. The downstream localization of Rac1 in the signaling cascade is further corroborated by the fact that the presence of WAVE-2, which is normally a downstream effector of Rac1 [52], at the leading edge was unchanged under these conditions. On the other hand, in the LPA-treated CR-*ACTG1* clones, there were as many mature FAs (α-parvin-rich) as there were in the CR-*ACTB* cells, but they were bigger. Moreover, there was a smaller number of nascent FAs (VASP-rich) in CR-*ACTG1* cells than in CR-*ACTB* cells. According to Wozniak and colleagues, Mena/VASP, together with Vinculin, act at nascent FAs (initial adhesions), whereas Src and FAK are recruited to adhesion sites later during the clustering of integrins [54]. Alternatively, at nascent FAs, kindlin, together with the integrin-linked kinase (ILK):LIM and senescent cell antigen-like-containing domain protein (PINCH):parvin complex [55] and/or the migfilin:VASP complex [56], associates with integrins at first and, following FAK and Src, associates with nascent FAs [55]. Here, however, we observed α-parvin’s presence at well-developed FAs. In accordance with the literature, FA formation activates RhoA [57], but FA size may not determine the range of RhoA activation, but instead the ability to form new FAs. However, the diminished activity of RhoA in CR-*ACTG1* cells might not be solely caused by the altered FA turnover. We also assume that the extent of stress fiber formation somehow influences RhoA activity in a negative feedback loop as the highest number of thickly bundled stress fibers and lowest RhoA activity, in comparison to control cells, were noted for CR-*ACTG1* cells. We want to stress our belief that, at the core, the observed phenotypes are probably due to the different properties of actin isoforms in terms of their interactions with ABPs. Interestingly, we did not observe any changes in Cdc42 activity in response to LPA or PMA stimulation, though there is an interplay between Rho-GTPases [58]. These would be in agreement with the notion that Rac1 and RhoA are mainly affected by the changes in dynamics of integrin-based structures [59] and here we observed disturbed FA formation/turnover.

We showed here that the studied actin isoforms have different effects on FA formation. Alterations in FA formation in cells lacking either β or γ actin were accompanied by alterations in FA-associated signaling. Coherently with our findings, others noted that in the cells isolated from *ACTB^-/-^*-mice, there were more stress fibers and FAs than in controls [41,60]. On the other hand, it was shown for HeLa cells that down-regulation of *ACTB* caused by the knockdown of the gene that encodes the RNA-binding protein HuR was accompanied by smaller numbers of stress fibers and impaired adhesion [61]. Additionally, in contrast to our findings, Bunnell with colleagues observed that enhanced FA formation was positively correlated with impaired 2-D migration in mouse embryonic fibroblasts devoid of β actin [41]. This could, however, be explained by the use of a different cellular model. Recently, we showed that A375 cells with lowered thymosin β4 expression exhibited a higher number of FAs and this was positively correlated with impaired 2-D and 3-D migration [62]. It is definitely worthwhile to continue to study the influence of actin isoforms on FA formation and composition as the integrins, receptors of extracellular matrix (ECM) proteins and key components of FAs, are being discussed as therapeutic targets in melanoma [63] and interact with receptor tyrosine kinases [52], like epidermal growth factor receptor (EGFR) or hepatocyte growth factor receptor (c-MET), which play an important role in melanoma cells (discussed in [64]). 

Certainly, adhesion is crucial for anchorage-dependent cell motility [52]. Because of that, we examined the motility of the tested clones. We showed earlier on mesenchymally and ameboidally moving cells [65,66] that β and γ actins have the same effect on the motility of the tested cell lines; however, our previous approach was based on isoactin overexpression and the endogenous presence of both isoactins in the cell could obscure the effect of overexpression. Here, in the wound-healing experiments, which mirror collective migration, we noted that CR-*ACTB* cells moved faster than control cells, whereas the CR-*ACTG1* clones closed the wound more slowly than CR-*ACTB* and control cells. These findings were in alignment with the observed lamellipodial activity of both types of clones upon PMA exposure. It was also previously shown that lowered γ actin levels in neuroblastoma cells resulted in slower collective migration [35]. However, in terms of invasiveness, the lack of both isoactins had diminishing effects, although the clones formed an increased number of invadopodia and they were functional in terms of ECM digestion. We hypothesize that both isoactins have to be present in invadopodia, probably due to the structural features of filamentous actin or recruitment of appropriate ABPs, since our earlier studies showed that both β and γ actin are present in active invadopodia [67]. Our invasion results differ from those obtained by Dugina and colleagues [34], which showed that reduced γ actin levels were accompanied by decreased invasion potential, but reduced β actin expression increased the invasion potential. This discrepancy can again be explained by different cellular models and, thus, divergent ABP landscapes and the fact that they used short hairpin RNA (shRNA) to down-regulate the expression of the given isoactin and here we had a complete knockout of non-muscle actin genes. Nevertheless, similarly to Dugina and colleagues [34], we observed that γ actin plays a more important role than β actin, at least in melanoma cells, in motility and adhesion. 

We believe that with this study, we placed another brick in our understanding of the complex interaction between actin cytoskeleton components. We are deeply convinced that our results clearly indicate that both non-muscle actins play only partly redundant roles in melanoma cells’ adhesion and motility and that changes in the β:γ actin ratio can reprogram a cell’s fate; if the functions of β and γ actin were exactly the same in melanoma cells, the lack of one of these non-muscle actins would be compensated for by elevations in the level of the other actin and thus result in restored cellular functions. Moreover, we were able to show that the expression level of each isoactin influences the expression of different sets of proteins. Having generated stable clones that were deprived of β or γ actin, it is crucial to address several pivotal issues in future studies. One such issue is the influence of knockout of *ACTB* or *ACTG1* on the cell proliferation rate, signaling pathways involved in tumor progression, and nuclear processes regulated by actin. Alas, it is of high priority to design studies to analyze, in depth, the nature of the interactions between non-muscle actins and ABPs, such as the Arp2/3 complex, VASP, FHOD1, or Myosin IIA, as the differences between the ABPs could be at least partially responsible for the aforementioned effects. On that note, a thorough knowledge of the mechanisms that regulate melanoma cells’ motility would allow the design of new cancer therapies.

## 4. Materials and Methods 

### 4.1. Materials

Plasmids used for inactivation of *ACTB* (catalogue number: sc-400000-NIC) and *ACTG1* (catalogue number: sc-400006-NIC) genes as well control set of plasmids (sc-437281), phorbol-12-myristate-13-acetate (PMA), lysophosphatidic acid (LPA), and cytochalasin D were bought at Santa Cruz Biotechnology Inc. (Heidelberg, Germany). Protease Inhibitors Cocktail, Phosphatase Inhibitor Cocktail 2, Phosphatase Inhibitor Cocktail 3, and Bradford reagent were from Merck (Darmstadt, Germany). Recombinant human β actin (04-CE15) was purchased from Novoprotein (Summit, NJ, USA), whereas recombinant human γ actin (TP301730) was from OriGene (Rockville, MD, USA). All other reagents were of analytical grade.

### 4.2. Cell Lines and Cell Culture Conditions

A375 and Hs294T cell lines were obtained from the American Type Culture Collection (LGC Standards, Lomianki, Poland), WM1341D and WM9 cell lines were from Rockland Immunochemicals, Inc. (Limerick, PA, USA), whereas SK-MEL-28 were bought at CLS Cell Lines Service GmbH (Eppelheim, Germany). Cell lines were subcultured twice a week. Some experiments were conducted on PMA- and LPA-stimulated cells. Before stimulations, cells were starved for 24 h in medium without FBS (fetal bovine serum). Cells were stimulated with 1 µM LPA for 10 min or with 100 nanomolar (nM) PMA for 5 min at 37 °C in 5% CO_2_.

### 4.3. CRISPR/Cas9(D10A) Inactivation of ACTB and ACTG1 Genes

A375 cells growing in a 35-mm plate were transfected with a mixture of two plasmids coding for Cas9(D10A), two specific for a gene of interest gRNAs (or scrambled gRNAs in the case of control clones), puromycin resistance, and green fluorescent protein (GFP), according to the manufacturer and with the help of Lipofectamine™ 2000 (ThermoFisher Scientific, Warsaw, Poland). The gRNAs’ sequences for *ACTB* are 5′atgtgcaaggccggcttcgc3′ and 5′gccgttgtcgacgacgagcg3′, and for *ACTG1* – 5′gcacccagtgctgctgaccg3′ and 5′cacgcgcagctcgttgtaga3′. Information about control gRNAs sequences is confidential, according to the manufacturer. Upon 24 h, the cells were trypsynized and seeded onto a 15-cm plate. On the next day, the medium was changed to selection medium containing 1 μg/mL of puromycin, which was next replenished every 2–3 days. After approximately two weeks of selection, single clones were isolated by the application of glass cylinders and silicone paste. From this moment every clone was cultivated further in the selection medium with 0.5 μg/mL puromycin. The clones were verified for the absence of β and γ actin by immunostaining, Western blot, qPCR, and gDNA analysis. We found out that upon selection of the clones there was no GFP signal. It could be caused by the fact that puromycin resistance and GFP were not coded by the same plasmid, which in turn caused most probably removal of the plasmid-encoding GFP from cells’ genome. 

### 4.4. Invasion Assay

The assay is described elsewhere [62]. Briefly, the assay was performed using Transwell™ filters (BD Biosciences, Wroclaw, Poland) coated with Matrigel™ (BD Biosciences, Wroclaw, Poland). Cells that traversed the Matrigel™ layer and attached to the lower surface of the filter were stained with Hoechst 33342 and counted under a fluorescent microscope. 

### 4.5. Cell Migration Assays

For spontaneous migration assay, 1000 cells were seeded into wells of 96-well IncuCyte ImageLock plates. Migration assay was performed using IncuCyte^®^ Live Cell Analysis Imaging System (Essen BioScience, Ltd., Royston, UK) for 72 h with images taken every 2 h. Using Manual Tracking plug-in (ImageJ, version 1.52p, F. Cordelieres, Institute Curie, Paris, France), cellular trajectory, velocity, and distance covered by a single cell were determined. End-point directionality was calculated as the ratio between straight-line displacement (distance to origin, DTO) and total path traveled by the cell (total distance, TD) [62]. For each parameters, 30 cells (10 cells per clone) per group were analyzed. 

For collective migration assay, cells were cultured in 96-well IncuCyte^®^ ImageLock plates. Upon reaching the confluence by the cells, wounds in the cells’ monolayers were done using IncuCyte^®^ WoundMaker. Then, the closure of the wound was followed using IncuCyte^®^ Live Cell Analysis Imaging System. Images collected every 2 h for 48 h were used for data analysis (IncuCyte^®^ ZOOM 2018A software, Essen BioScience, Ltd., Royston, UK), which was presented as a percent of scratch overgrown by the cells over time. 

### 4.6. Immunocytochemistry and Confocal Microscopy

For immunostaining assays, the cells cultured on glass coverslips were fixed with 4% formaldehyde for 20 min and then permeabilized with 0.1% Triton X-100 in phosphate-buffered saline (PBS). Nonspecific bindings were blocked by using 1% bovine serum albumin (BSA) in PBS. The coverslips were incubated overnight at 4 °C with primary antibodies diluted in blocking solution. Dilutions are shown in Table S1. Depending on the experiment, the coverslips were incubated with secondary antibodies conjugated with different Alexa-Fluor™ dyes (Table S1) and with Hoechst 33342 (ThermoFisher Scientific, Warsaw, Poland) to stain cells’ nuclei. In order to detect G-actin and F-actin, some stainings were done with the usage of Alexa Fluor™ 594-labeled DNase I and Alexa Fluor™ 488-labeled phalloidin, respectively. Both were from ThermoFisher Scientific (Warsaw, Poland) and diluted at 1:100. At the end, the coverslips were mounted on glass slides with Dako Mounting Medium (Agilent Technologies Inc., Santa Clara, CA, USA). Photos were taken and analyzed with Leica TCS SP8 Confocal Laser Scanning Microscope and Leica Application Suite X (LAS X). Some of the microphotographs are presented as negatives of grayscale mode to better present the details of the stainings. 

Plot profiles. Distribution of proteins across the cells was presented as a mean of fluorescence intensity expressed in arbitrary units (AU) along the straight line profiles (µm). Lines were created manually with quantify tools of Leica Application Suite X (LAS X). Fluorescence intensity was then automatically generated for each channel separately. Photos were taken at the same settings. Nine cells per group were analyzed. 

Area under curve graph was created by using Graphpad Prism 8 based on the leading edge dynamics’ data. Results were presented as a fold change normalized to the CR-CTRL group. 

### 4.7. Live Imaging and Kymograph Analysis

The cells were seeded onto Nunc™ Lab-Tek™ Chambered Coverglass (ThermoFisher Scientific, Warsaw, Poland) and on the following day transfected with pCMV-LifeAct-TagRFP (ibidi GmbH, Gräfelfing, Germany) with the help of Lipofectamine™ 3000 reagent (ThermoFisher Scientific, Warsaw, Poland). On the next day, the medium was changed to medium without FBS. Then, 24 h later, the cells were stimulated with 100 nM PMA and observed with the help of Leica TCS SP8 Confocal Laser Scanning Microscope. Pictures were taken every 10 s for 5 min prior to stimulation and 10 min after addition of PMA. Obtained movies were then analyzed with ImageJ software (version 1.52p, F. Cordelieres, Institute Curie, Paris, France). Kymographs were generated along 5-pixel-wide line regions oriented in the direction of individual protrusions. Quantitative data about the leading edge position were then exported to Excel and finally area under curve (AUC) was calculated and presented as bars for every condition (*n* = 6).

### 4.8. Western Blot Analysis

The cell lysates were prepared by scraping the cells on ice in cytoskeletal-bound protein extraction buffer (10 mM Tris-HCl pH 7.4, 100 mM NaCl, 1 mM ethylenediaminetetraacetic acid (EDTA), 1 mM ethylene glycol-bis(2-aminoethylether)-N,N,N′,N′-tetraacetic acid (EGTA), 1 mM NaF, 20 mM Na_4_P_2_O_7_, 2 mM Na_3_VO_4_, 1% Triton X-100, 10% glycerol, 0.1% SDS, 0.5% sodium deoxycholate) or 2-D electrophoresis cell lysis buffer (7 M urea, 2 M thiourea, 4% 3-[(3-cholamidopropyl)dimethylammonio]-1-propanesulfonate hydrate (CHAPS), 50 mM Dithiothreitol, 0.2% BioLyte^®^ 3–10, 1:100 serine phosphatase inhibitor, 1:100 tyrosine phosphatase inhibitor), both with addition of 1:100 protease inhibitor cocktail. The protein concentration was established by using the Bradford protein assay. The samples containing forty μg of protein were run on either 12.5% or 15% polyacrylamide gel by SDS-PAGE and transferred to nitrocellulose by wet transfer. The efficiency of the transfer and equal protein loading were determined by using the Ponceau S staining of the membrane. The membranes were blocked as next in 5% skimmed milk or 5% bovine serum albumin in Tris-buffered saline with 0.1% Tween 20 (TBS-T), depending on antibody manufacturer’s recommendations. The rest of the procedure is described elsewhere [62]. The densitometric analysis was performed using the Image Lab software (Bio-Rad). The bands were standardized to whole protein content in analyzed lane (Ponceau S) and then normalized against mean of protein expression in the control group.

Stain-free gel (Mini-PROTEAN^®^ TGX Stain-Free™ Precast Gel) (Figure S2) was bought at Bio-Rad (Hercules, CA, USA). The proteins upon SDS-PAGE were visualized in the “stain-free modus” with the help of the ChemiDoc MP System and Image Lab software (version 5.2.1, Bio-Rad, Hercules, CA, USA).

### 4.9. Quantitative Polymerase Chain Reaction (qPCR) 

RNA was isolated with GenElute^TM^ Mammalian Total RNA Miniprep Kit (Merck, Darmstadt, Germany). Next, RNA was treated with DNase I (Merck, Darmstadt, Germany) and reversely transcribed using High Capacity cDNA Reverse Transcription Kit (ThermoFisher Scientific, Warsaw, Poland), following the manufacturer’s instructions. Real-time PCR reactions were carried out using the PowerUp™ SYBR™ Green Master Mix (ThermoFisher Scientific, Warsaw, Poland), in accordance to the seller’s recommendations on StepOnePlus Real-Time PCR Systems device (ThermoFisher Scientific, Warsaw, Poland). The primers are listed in Table S2. Results were normalized against expression of *HPRT1*.

### 4.10. 2-D PAGE

Proteins were extracted from A375 cells in 2-D electrophoresis cell lysis buffer (7 M urea, 2 M thiourea, 4% CHAPS, 50 mM Dithiothreitol, 0.2% BioLyte^®^ 3–10, 1:100 Phosphatase Inhibitor Cocktail 2, 1:100 Phosphatase Inhibitor Cocktail 3). A sample containing twenty μg of protein was run for 2-D PAGE using ReadyStrip™ IPG Strips pH 4–7 (Bio-Rad, Hercules, CA, USA) for the first dimension and following for second dimension on 12.5% SDS-PAGE. Finally, the gel was stained with Deep Silver Kit (MoBiTec GmbH, Göttingen, Germany), according to the manufacturer’s instructions. The photo was taken with the ChemiDoc MP System (Bio-Rad, Hercules, CA, USA).

### 4.11. Rho-GTPases Assays

G-LISA Activation Assays (Cytoskeleton, Inc., Denver, CO, USA) were used to compare the levels of activated Rac1 (BK128), RhoA (BK124), and Cdc42 (BK127) GTPases in different groups of cells after LPA and PMA stimulations. Lysates were prepared according to the protocol, except for lysates for G-LISA Cdc42 Activation Assay, in which lysis buffer was a mixture of GL35:GL36 buffers in ratio 1:1. This was done upon an advice of technical support of Cytoskeleton, Inc. The Precision Red Protein Assay reagent was used for quantification of protein concentration in lysates, which was around 0.5 mg/mL. Active proteins were detected by incubation with primary antibody detecting given GTPase and appropriate secondary antibody conjugated with horseradish peroxidase (HRP). Colorimetric signal was measured at 490 nm using a microplate spectrophotometer. The data were presented as ratio between stimulated and nonstimulated (starved for 24 h) cells with normalization to the control cells.

### 4.12. F:G Actin Ratio Assay

Cells were harvested and lysed in actin stabilizing buffer (50 mM 1,4-piperazinediethanesulfonic acid (PIPES) pH 6.9; 50 mM NaCl; 5 mM MgCl_2_; 5 mM EGTA; 5% glycerol; 0.1% Nonidet P40; 0.1% Triton X-100; 0.1% Tween 20; 0.1% 2-mercaptoethanol; 0.001% AntifoamC; cocktail of protease inhibitors 1:100; 1 mM ATP). Lysates were incubated for 10 min at 37 °C and centrifuged 100–300× *g* for 5 min (elimination of nonlysed cells, plasma membranes, and cellular organelles). Supernatants were centrifuged for 60 min at 100,000× *g* in Optima MAX-XP with TLA-55 rotor to separate F-actin from G-actin. G-actin was present in supernatant and F-actin in pellet. Supernatants were collected, while pellets were resuspended in actin depolymerization solution (10 μM cytochalasin D in water), and incubated for 1 h with occasional vortexing. Both fractions were analyzed using SDS-PAGE and Western blot. One-fifth of G-actin and F-actin fractions were loaded on lanes. Densitometric analysis was done as it is described in the Western blot analysis section. 

### 4.13. Focal Adhesion and Cell Area Analysis

The number of focal adhesions for untreated, PMA-, or LPA-stimulated cells was counted manually using annotation tools of LasX application, according to immunostaining for α-parvin or VASP. The data are presented as a mean FAs’ number per cell. FAs’ and cell area were marked manually and measured automatically by using “Analyze > measure” command in ImageJ software (version 1.52p, F. Cordelieres, Institute Curie, Paris, France) (with previous scale settings). In the case of FAs’ number and cell area, 30 cells per group were analyzed. Then, area of FAs presents in nine cells chosen from earlier analyzed pool of cells were measured, which gave us the opportunity to observe the potential correlation between the number and size of FAs.

### 4.14. Lamellipodium Width and Thickness Analysis

All analyses were done using the ZEISS LSM 510 confocal microscope (Zeiss, Oberkochen, Germany) and dedicated to this microscope software. The cells were seeded onto glass coverslips, treated with 100 nM PMA for 5 min, and stained with phalloidin-Alexa Fluor™ 488 and DNase I-Alexa Fluor™ 568. The images of immunofluorescent staining were analyzed within a clone by choosing approx. 10 cells per clone in different planes of sight. The width of lamellipodium was measured by guiding a histogram through the lamellipodium using a ruler in the software. The thickness of the lamellipodium was calculated by guiding a histogram through the lamellipodium and reading the whole intensity of fluorescence through the lamellipodium.

### 4.15. Stress Fibers’ Formation Analysis

The analysis was done using the ZEISS LSM 510 confocal microscope (Zeiss, Oberkochen, Germany) and dedicated to this microscope software. The cells were seeded onto glass coverslips and, after 24 h, treated with 1 μM LPA for 10 min. Staining and choosing of cells for analysis was done as described in the paragraph above. The number of thickly bundled stress fibers per cell was assessed by guiding a histogram through the width of the cell containing thick stress fibers and identifying the number of fluorescent values that were higher or equal to 75% of the highest fluorescence peak.

### 4.16. Gelatin Digestion Assay 

The assay was conducted in order to assess the number of active invadopodia in the cells. Poly-L-lysine coated coverslips (Corning, Corning, NY, USA) placed in a 24-well plate were rinsed with 1xPBS. Then the coverslips were fixed by incubation with 0.5% glutaraldehyde for 15 min at room temperature (RT), followed by another washing with PBS. The coverslips were then inverted onto a drop of fluorescein-conjugated gelatin solution (ThermoFisher Scientific, Warsaw, Poland) and incubated in the dark for 10 min at RT. After that, the coverslips were again inverted with the gelatin-coated side up and the residual reactive groups were quenched by adding 5 mg/mL sodium borohydride and 1 min incubation at RT, followed by washing with PBS. Before seeding the cells, the coverslips were rinsed with warm cell culture medium. The cells were seeded at the density of 30,000 per coverslip in a 24-well plate in full cell culture medium and kept at 37 °C, 5% CO_2_ for 12 h before fixing with 4% FA. The cells were stained using phalloidin-Alexa Fluor™ 568 and Hoechst 33342 (ThermoFisher Scientific, Warsaw, Poland) to detect F-actin and cell nuclei, respectively. The images were obtained by using confocal microscopy. The number of invadopodia and the area of gelatin digestion was estimated by using the ImageJ software (version 1.52p, F. Cordelieres, Institute Curie, Paris, France). A total number of 10 cells per clone were analyzed.

### 4.17. Statistical Analysis

For each analysis, groups consisting of 3 clones (three biological replicates) were analyzed. Statistical analysis and graphs were performed with the use of GraphPad Prism 7 and 8 (GraphPad Software Inc., San Diego, CA, USA). Normality distribution was analyzed with Shapiro–Wilks and D’Agostino–Pearson normality tests. Statistical significances of analyzed data were determined using the parametric or nonparametric versions of unpaired student’s t-test or ANOVA (one-way or two-way) tests, properly to obtained data set. The significance levels were set at *p* < 0.05 (*), *p* < 0.01 (**), *p* < 0.001 (***), and *p* < 0.0001 (****). All data were expressed as means ± standard deviations (SD) or ± standard of the mean (SEM) or median.

## Supplementary Materials

Supplementary materials can be found at https://www.mdpi.com/1422-0067/21/8/2746/s1.

## Abbreviations

ABPs	Actin-binding proteins
*ACTB*	gene coding for β actin
*ACTG1*	gene coding for γ actin
CR-CTRL	control cells
CR-*ACTB*	cells with knocked out *ACTB*
CR-*ACTG1*	cells with knocked out *ACTG1*
F-actin	filamentous, polymerized actin
G-actin	globular, monomeric actin
FA	focal adhesion
LPA	lysophosphatidic acid
PMA	phorbol-12-myristate-13-acetate
nFA	nascent focal adhesion
