# Supplementary material for: Knockout of ACTB and ACTG1 with CRISPR/Cas9(D10A) Technique Shows that Non-Muscle β and γ Actin Are Not Equal in Relation to Human Melanoma Cells’ Motility and Focal Adhesion Formation
Source: Int J Mol Sci. 2020 Apr 15;21(8):2746. doi: 10.3390/ijms21082746 (PMC7216121; doi:10.3390/ijms21082746)
Supplement: Supplementary file 1 [file ijms-21-02746-s001.pdf]

Knockout of *ACTB* and *ACTG1* with CRISPR/Cas9(D10A) Technique Shows that Non-Muscle β and γ Actin Are Not Equal in Relation to Human Melanoma Cells' Motility and Focal Adhesion Formation

Natalia Malek †, Ewa Mrówczyńska †, Aleksandra Michrowska, Ewa Mazurkiewicz, Iuliia Pavlyk and Antonina Joanna Mazur *

Department of Cell Pathology, Faculty of Biotechnology, University of Wroclaw, 50-383, Poland; en.malek@gmail.com (N.M); ewa.mrowczynska@uwr.edu.pl (E.M.); 282709@uwr.edu.pl (A.M.); ewa.mazurkiewicz@uwr.edu.pl (E.M.); pavlykj@gmail.com (I.P.)

* Correspondence: antonina.mazur@uwr.edu.pl ; Tel.: +48-713-756-206

† Authors contributed equally to the manuscript

Supplementary method

gDNA analysis

Genomic DNA was isolated with the help of DNA Purification Kit (EurX®,Gdansk, Poland) according to the manufacturer's instructions. PCR reaction was performed with Color Taq PCR Master Mix (2x) (EurX®,Gdansk, Poland) and primers listed in Table S2 annealing to the sequences upstream and downstream from the sequences recognized by guide RNAs coded by CRISPR/Cas9(D10A) plasmids. PCR products were analyzed in 2% agarose gel in Tris–acetate–EDTA (TAE) buffer. PCR products length of clones with knock out of actins isoforms were compared to PCR products length of control clones.

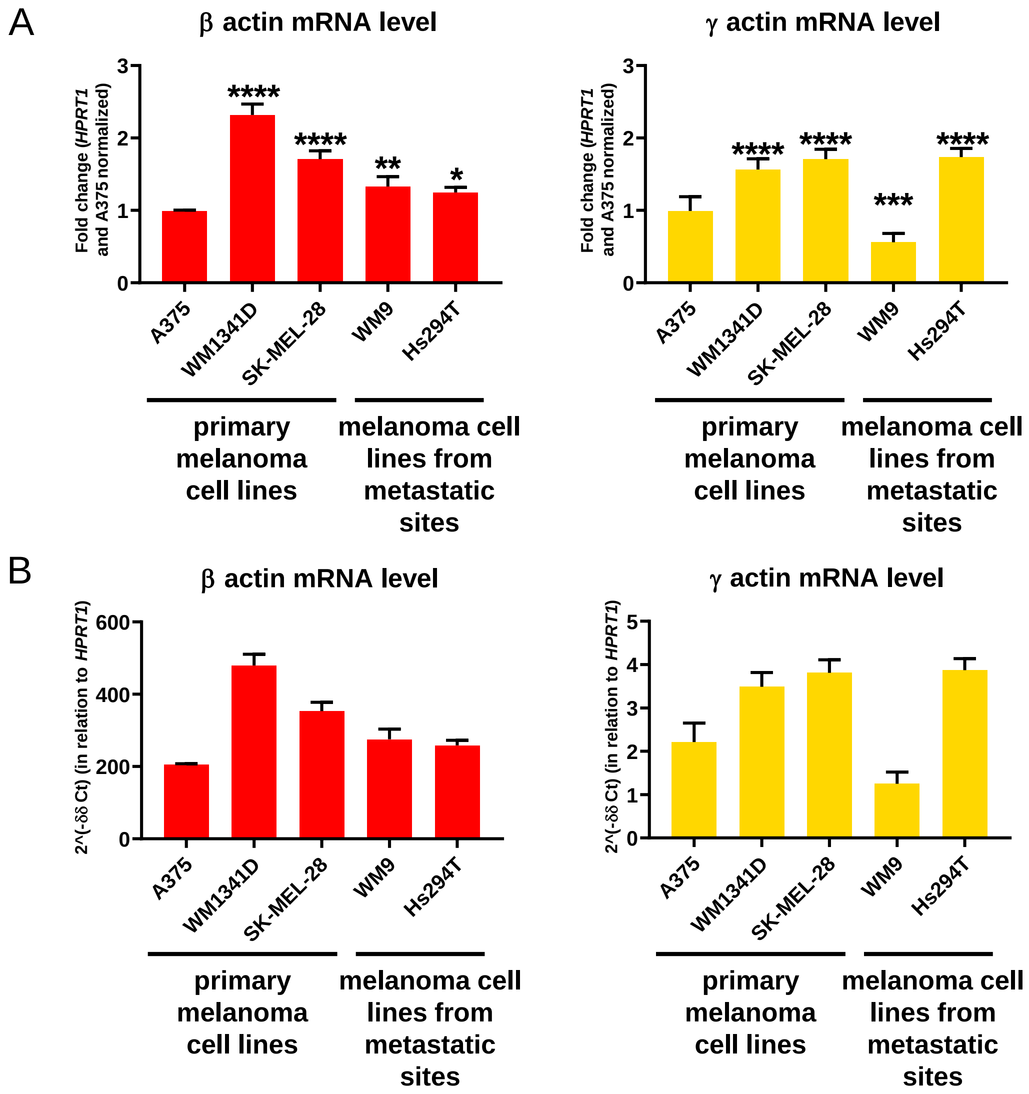

Figure S1. Quantitative RT-PCR analysis of *ACTB* and *ACTG1* expression levels. As templates served cDNAs of 5 melanoma cell lines. Results were normalized against *HPRT1* gene and A375 cells (n=3) (**A**) or only to *HPRT1* gene (**B**). Results are expressed as the mean ±SD. $p \leq 0.05$ (*), $p \leq 0.01$ (**), $p \leq 0.001$ (***), $p \leq 0.0001$ (****).

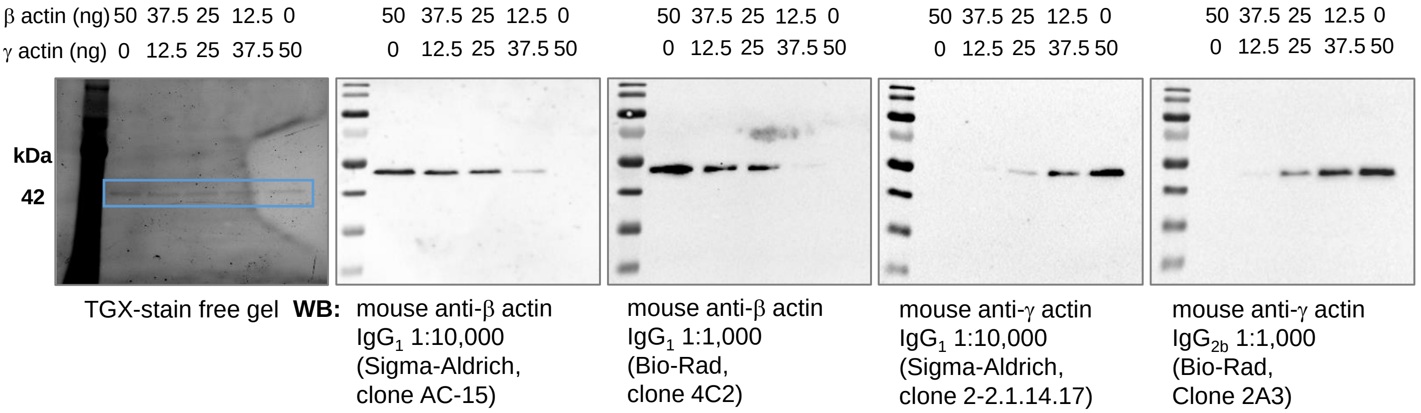

Figure S2. Analysis of the specificity of anti-β or γ actin antibodies used in this study. On the lanes different amounts of recombinant β or γ actin were applied as it is indiciated in the figure. Upon SDS-PAGE proteins were transfered to nitrocellulose membranes, which were probed with appropriate antibodies. Blue rectangle marks the actin band on a TGX-stain free gel. These antibodies were indistinguishably used in this study. Only for simultaneous immunofluorescent stainings to detect β and γ actin we used mouse anti-β actin IgG_1 (Bio-Rad) and mouse anti-γ actin IgG_{2b} (Bio-Rad) antibodies.

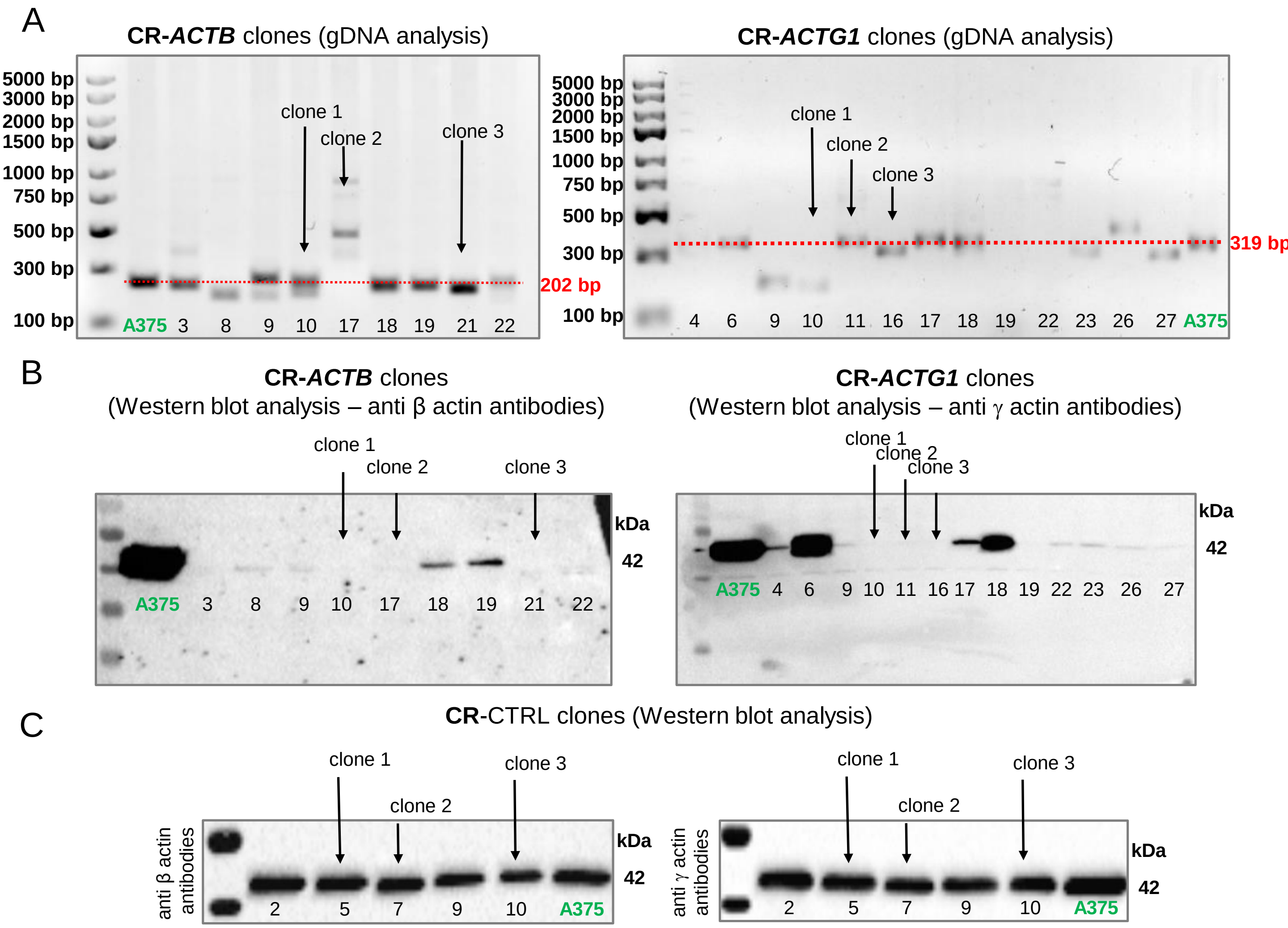

Figure S3. (**A**) Analysis of gDNA of obtained clones devoid of β or γ actin. Upon isolation of gDNA from clones PCR reactions were performed with appropriate starters and 100 ng of gDNA as a template. Products of reactions were analyzed in 2% TAE agarose gel. Western blot analysis of CR-*ACTB* and CR-*ACTG1* clones (**B**) and control clones (**C**) obtained upon CRISPR/Cas9(D10A) procedure. Membranes were probed with anti-β or γ actin antibodies. 40 µg of protein was loaded on every lane. bp – base pairs.

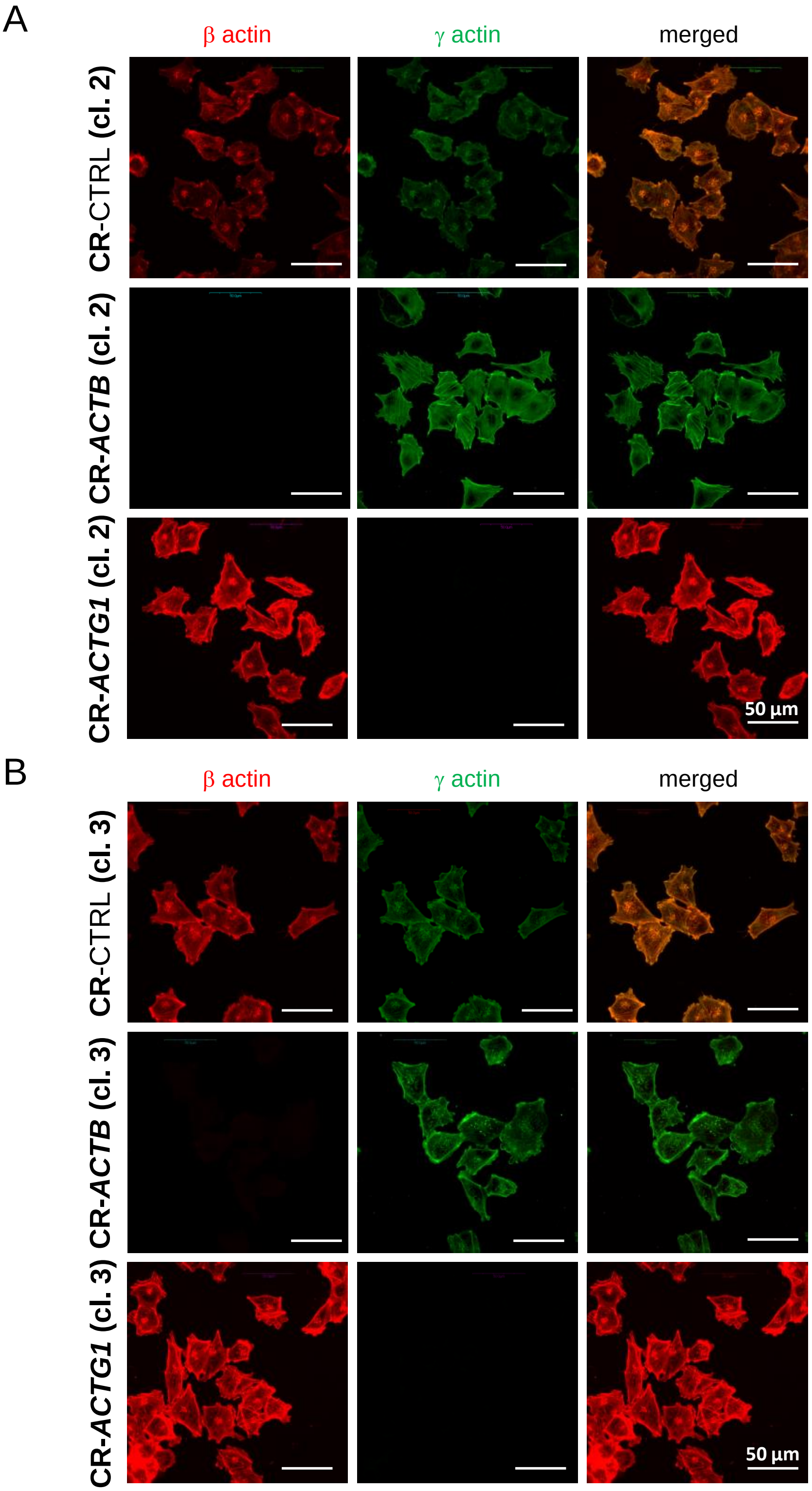

Figure S4. Immunocytochemical staining of control clones and clones devoid of β or γ actin. The cells were incubated with antibodies recognizing β and γ actin. Pictures were taken at the same settings during the same session at the microscope. Second (**A**) and third (**B**) part of clones.

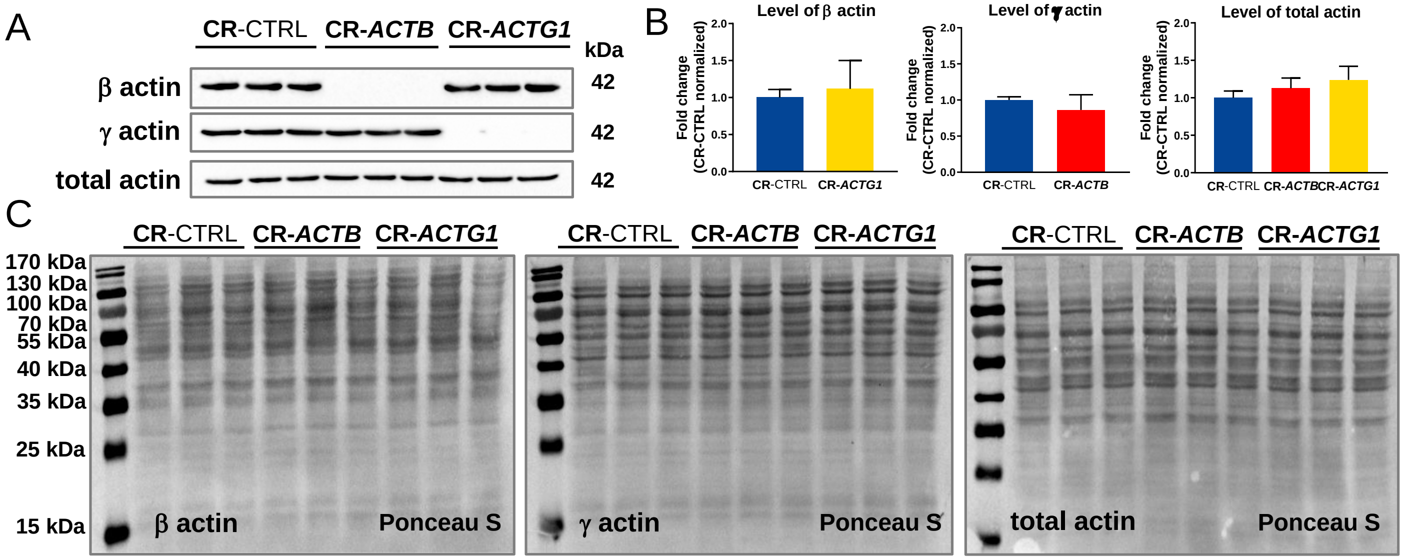

Figure S5. (**A**) Western blot analysis of cell lysates of CR-CTRL, CR-*ACTB* and CR-*ACTG1* clones. To detect the bands nitrocellulose sheets were probed with antibodies directed against β or γ actin and total actin. Forty µg of protein was loaded on every lane. Corresponding Ponceau S stainings of membranes are shown in **C**. (**B**) Densitometric analysis of WBs shown in A (n=3). Results are expressed as the mean ±SD.

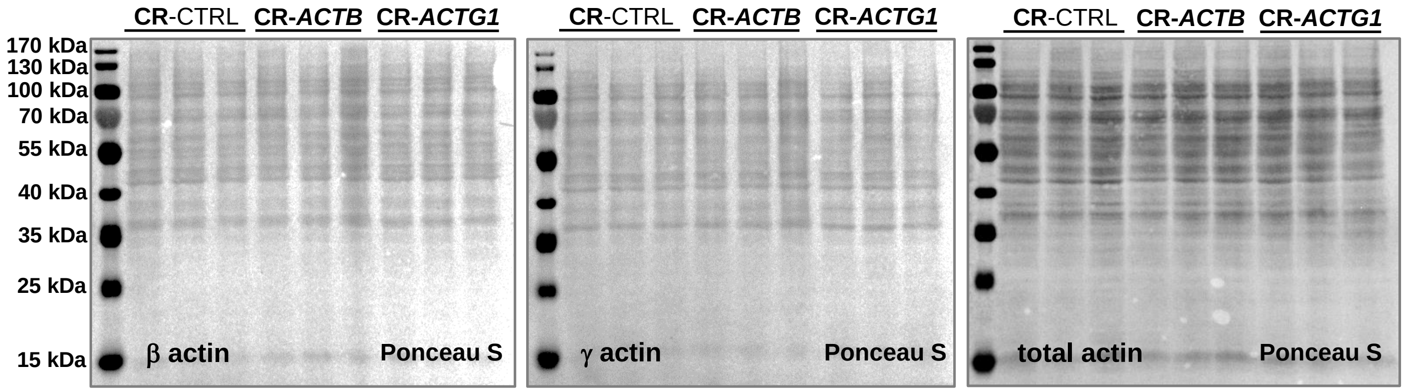

Figure S6. Corresponding Ponceau S stainings of membranes shown in Figures 2A.

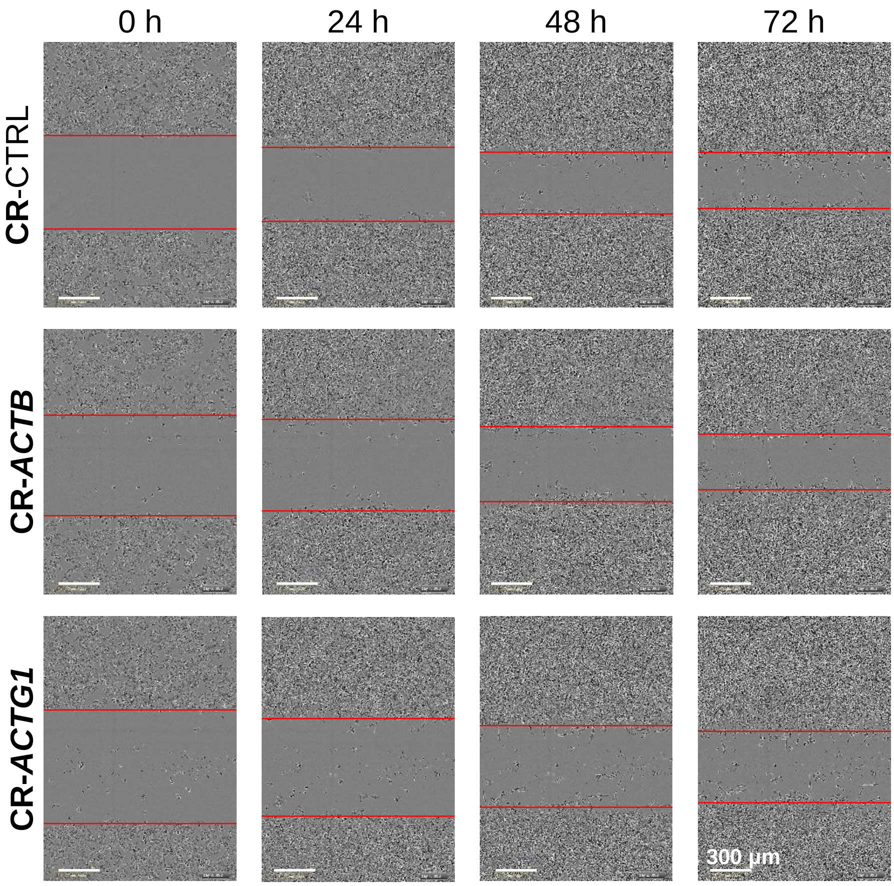

Figure S7. Representative pictures of cells migrating collectively recorded over 72 h for studied cells (n=3).

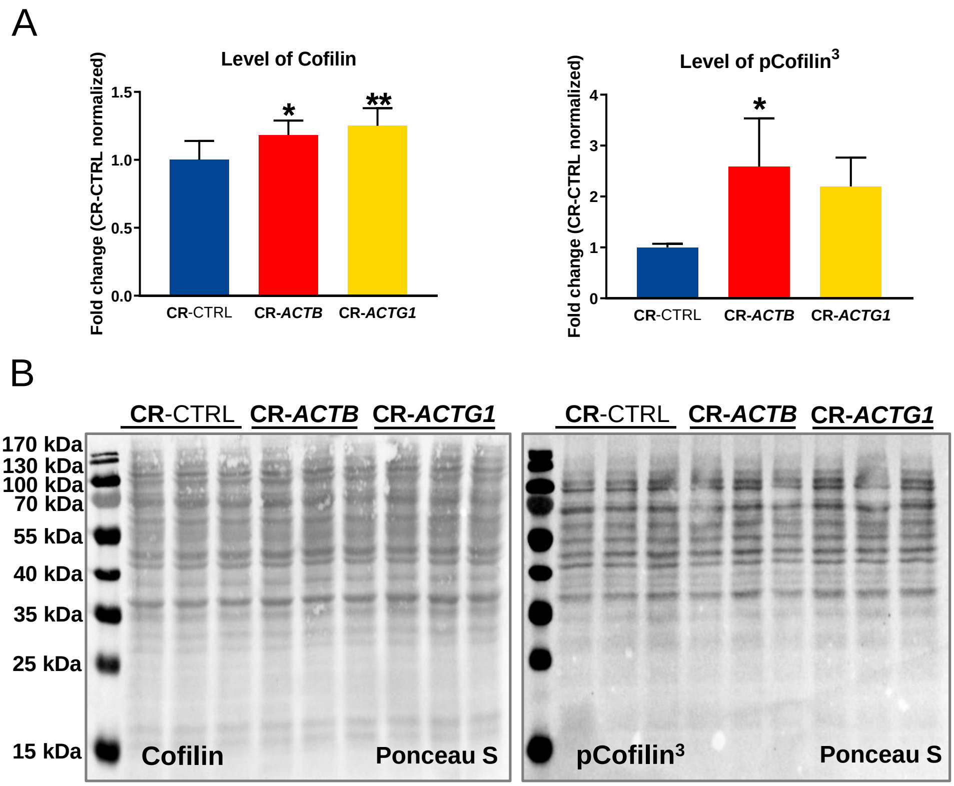

Figure S8. (**A**) Densitometric analysis of levels of Cofilin and pCofilin[3] analyzed with the help of Western blot procedure and shown in Fig. 3K (n=3-6). Results are expressed as the mean ±SD. $p \leq 0.05$ (*), $p \leq 0.01$ (**). Corresponding Ponceau S stainings of membranes are shown in **B**.

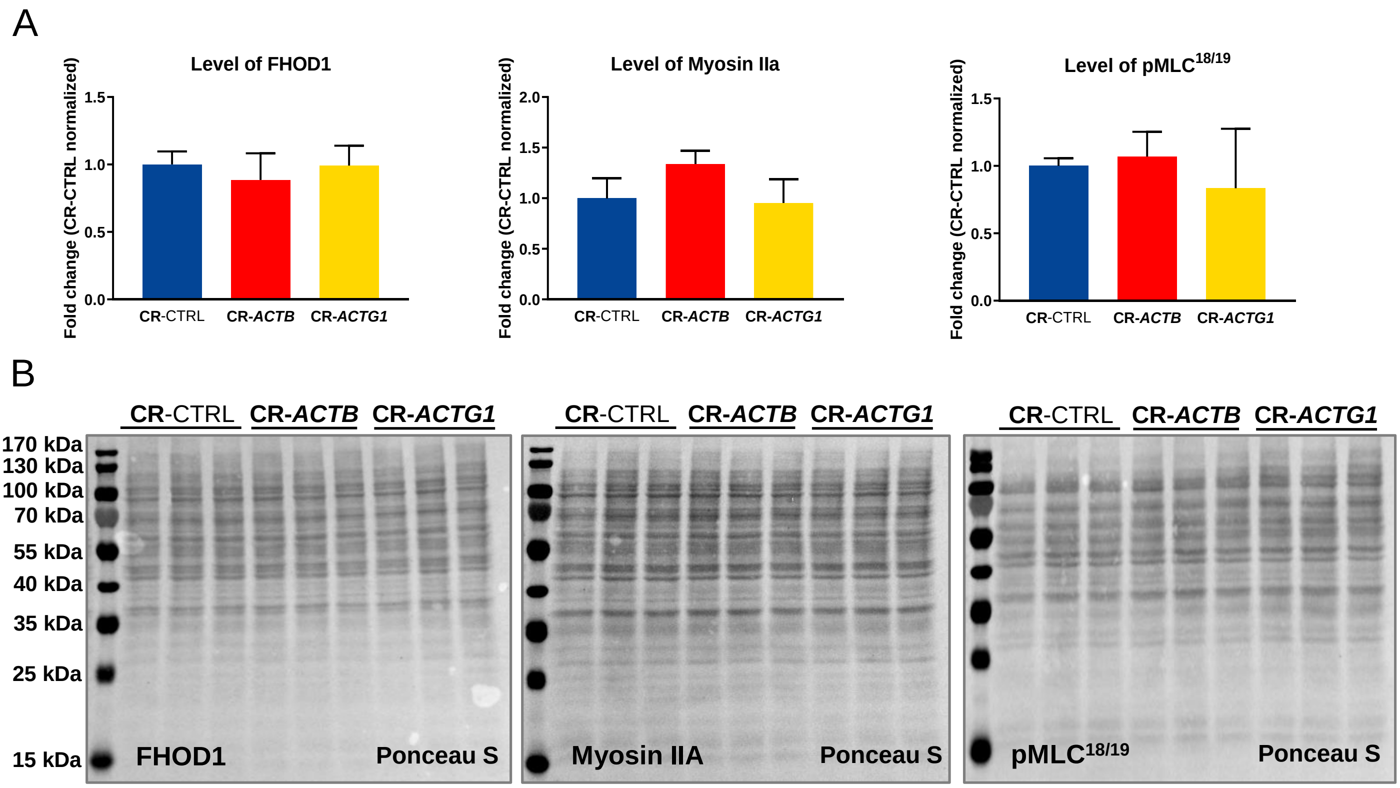

Figure S9. (**A**) Densitometric analysis of levels of FHOD1, Myosin IIA and pMLC$^{18/19}$ analyzed with the help of Western blot procedure and shown in Fig. 4G (n=3-6). Results are expressed as the mean ±SD. Corresponding Ponceau S stainings of membranes are shown in **B**.

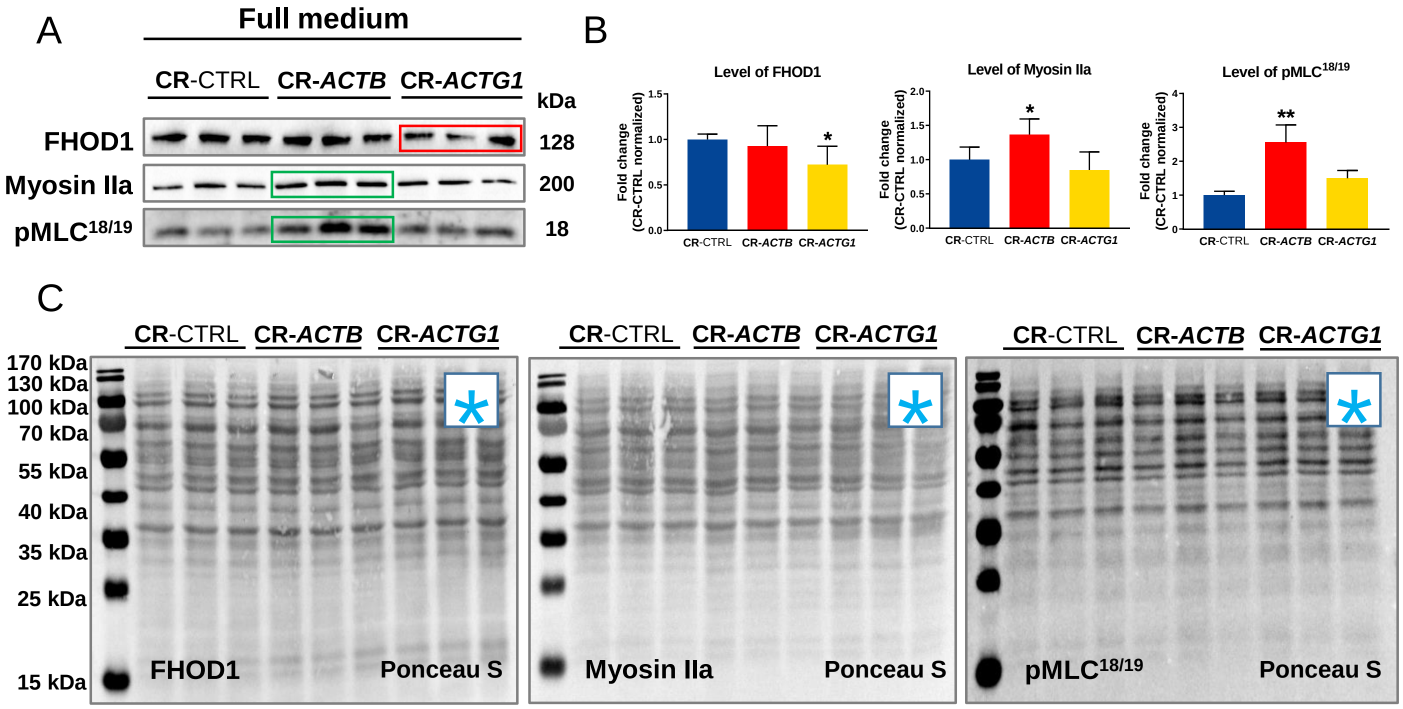

Figure S10. (**A**) Western blot analysis of cell lysates of clones growing in full medium. 40 μg of protein was loaded on every lane. The membranes were probed with antibodies recognizing FHOD1, Myosin IIa, and pMLC$^{18/19}$. Corresponding densitometric analysis is shown in **B** (n=3-6) and Ponceau S stainings of membranes are shown in **C**. Green and red rectangles represent statistically higher and lower level of protein, respectively, in comparison to control cells. Results are expressed as the mean ±SD. $p \leq 0.05$ (*), $p \leq 0.01$ (**). Blue asterics denote the membranes which were probed more than once, hence in this supplementary file they are presented more than one time.

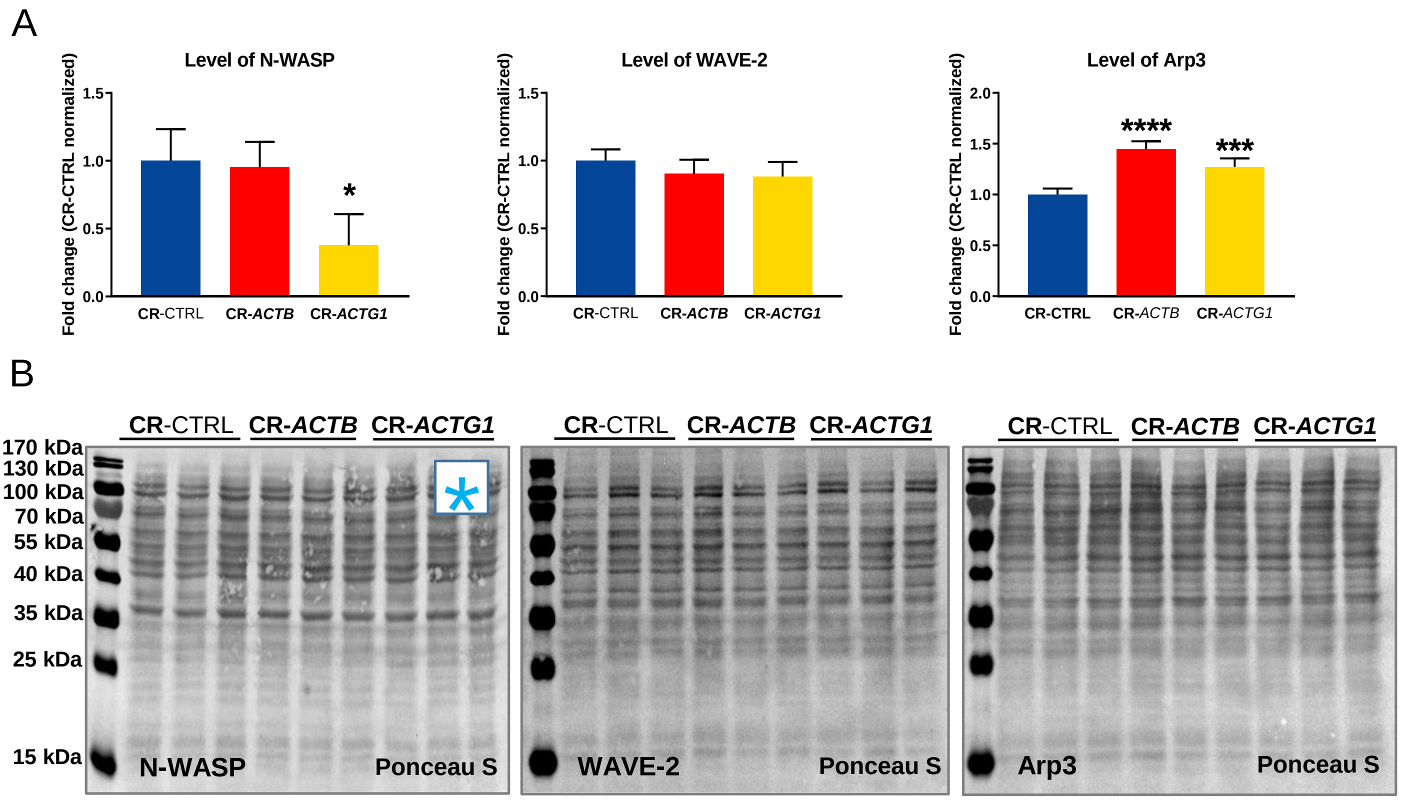

Figure S11. (**A**) Densitometric analysis of levels of N-WASP, WAVE-2 and Arp3 analyzed with the help of Western blot procedure and shown in Fig. 6E (n=3-6). Results are expressed as the mean ±SD. p ≤ 0.05 (*), p ≤ 0.01 (**), p ≤ 0.001 (***), p ≤ 0.0001 (****). Corresponding Ponceau S stainings of membranes are shown in **B**.

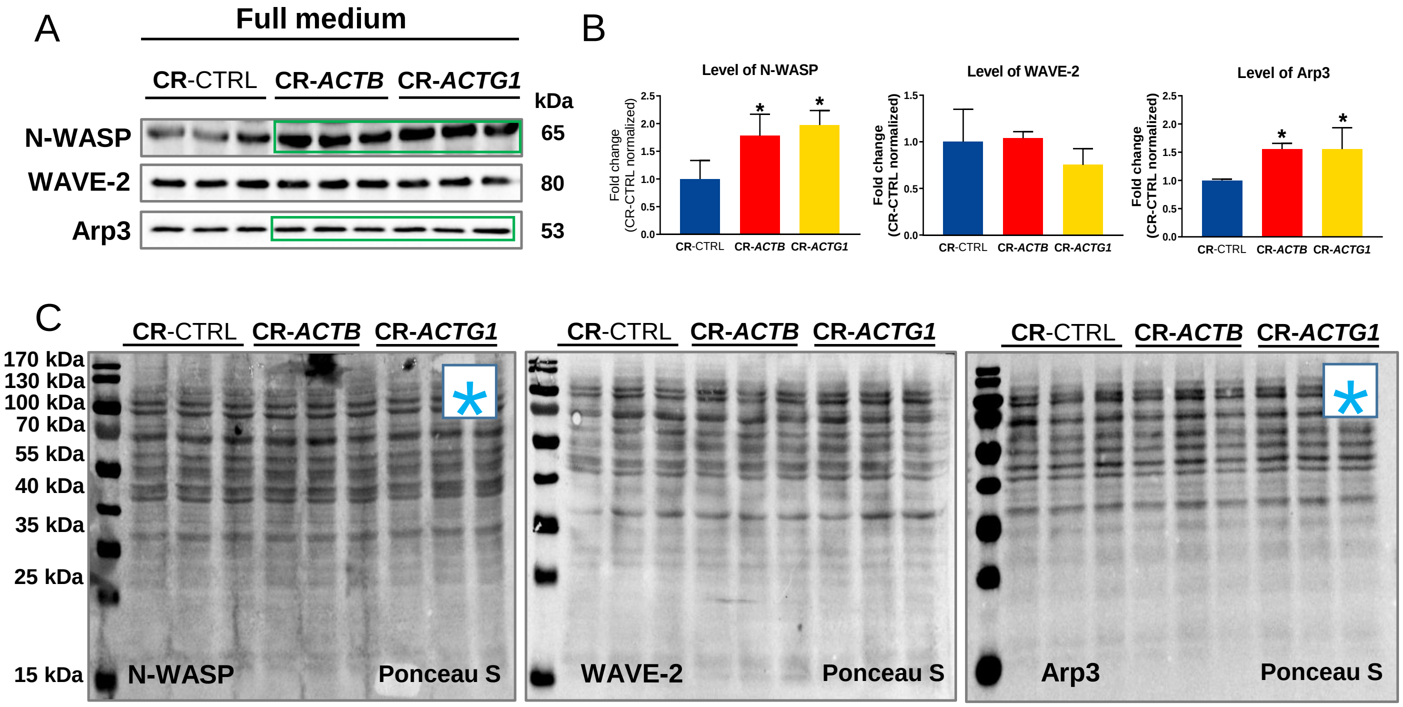

Figure S12. (**A**) Western blot analysis of cell lysates of clones growing in full medium. Forty µg of protein was loaded on every lane. The membranes were probed with antibodies recognizing N-WASP, WAVE-2 and Arp3. Corresponding densitometric analysis is shown in **B** (n=3) and Ponceau S stainings of membranes are shown in **C**. Green rectangles represent statistically higher level of protein in comparison to control cells. Results are expressed as the mean ±SD. p ≤ 0.05 (*). Blue asterics denote the membranes which were probed more than once, hence in this supplementary file they are presented more than one time.

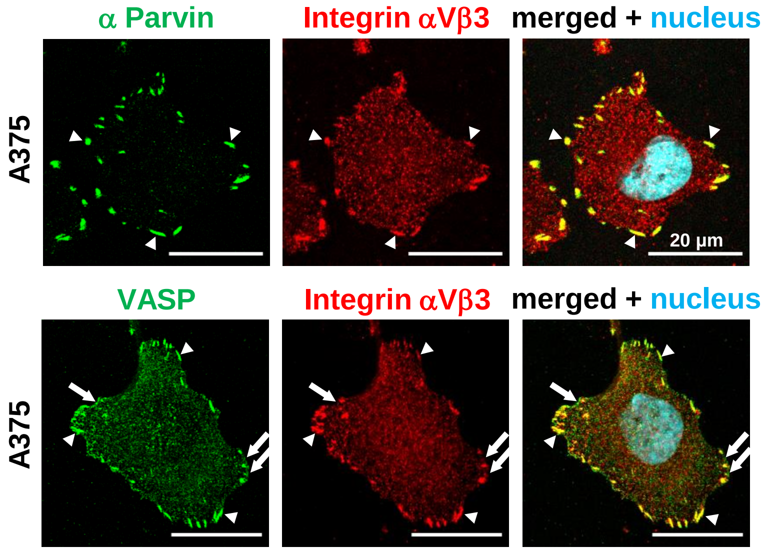

Figure S13. A375 cells were subjected to immunocytochemical analysis. Antibodies directed against α Parvin, VASP and integrin αVβ3 were used. White arrows point at accumulation of VASP and white arrowheads highlight focal adhesions. The cells were grown under full medium conditions.

Full medium

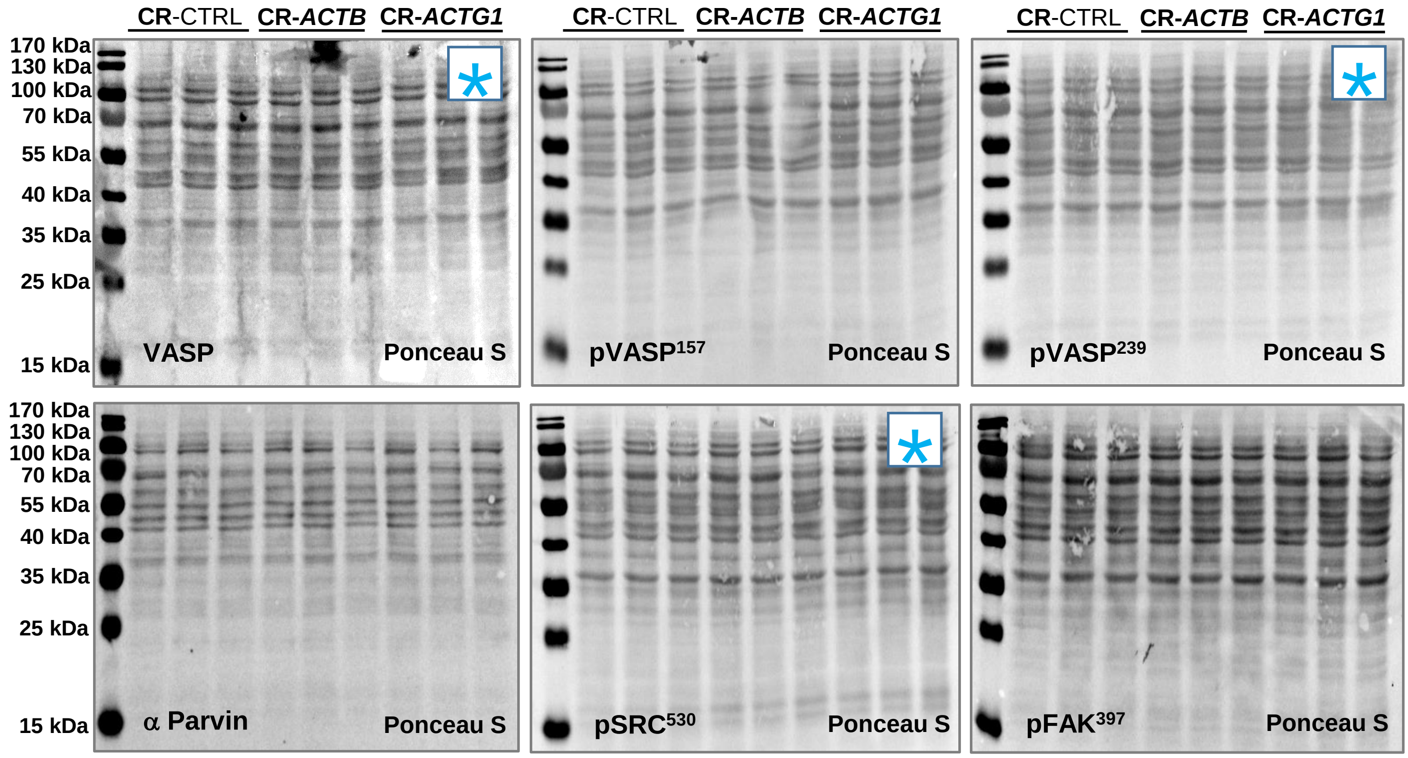

LPA 1 µM, 10’

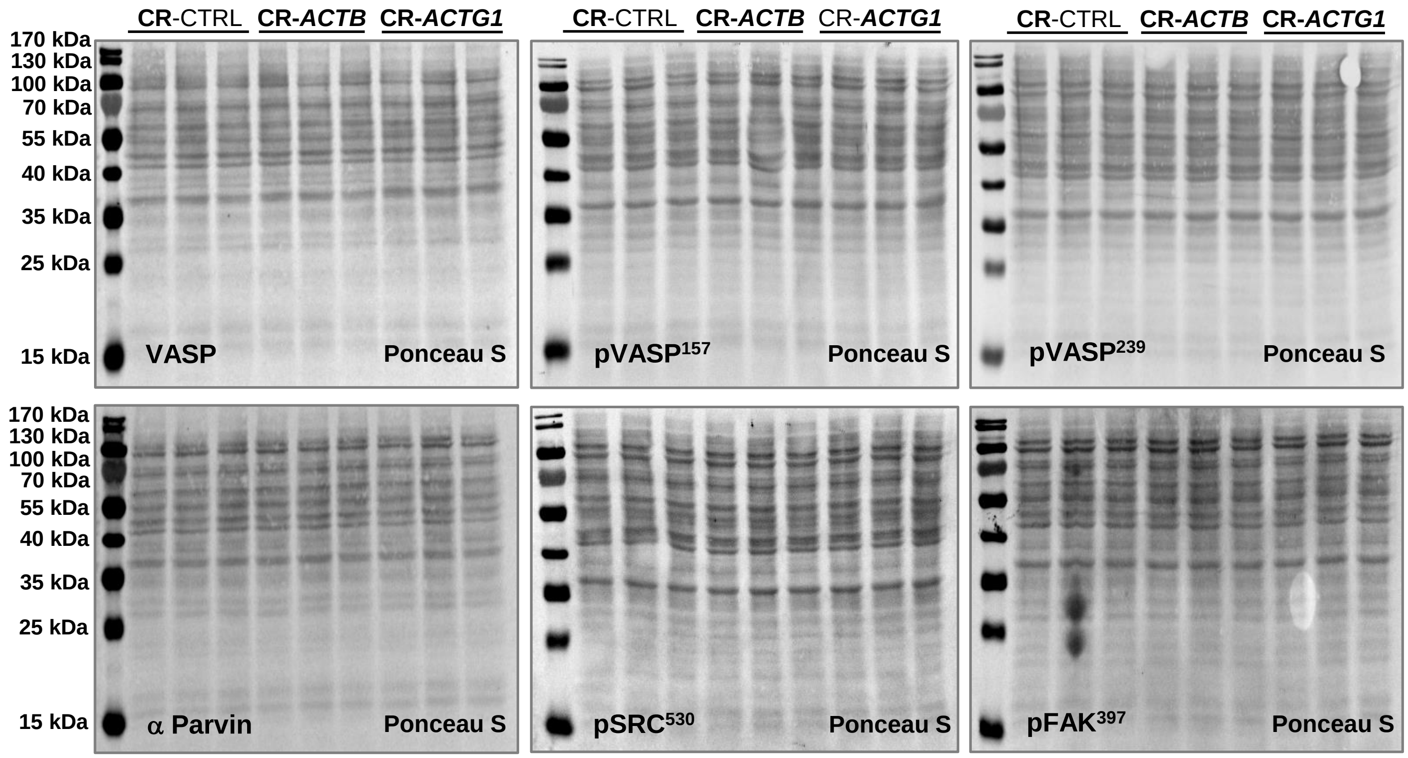

To be continued

PMA 100 nM, 5’

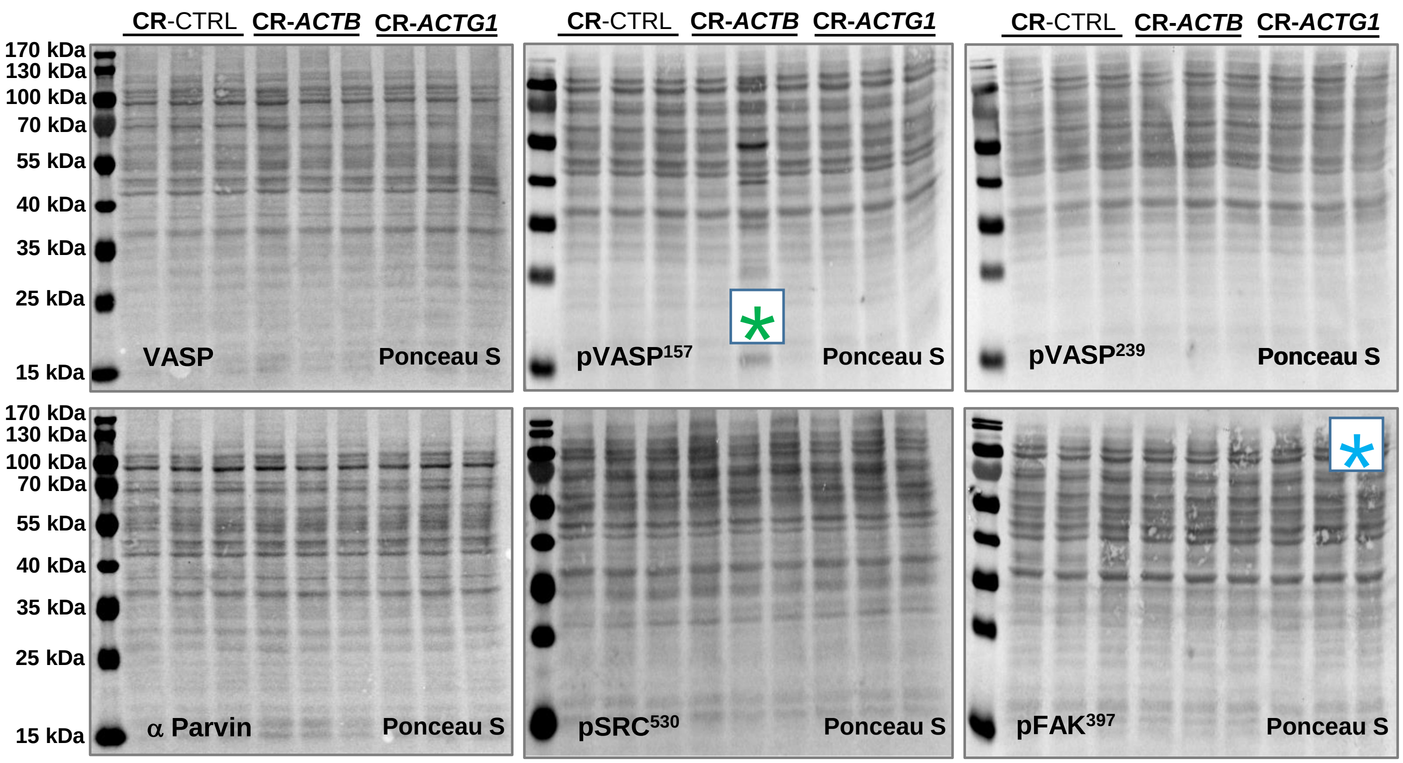

Figure S14. Corresponding Ponceau S stainings of membranes shown in Figure 9. Blue asterics denote the membranes which were probed more than once, hence in this supplementary file they are presented more than one time. Green asterics point at a lane on which accidentally 2 µl of Protein lader was loaded.

Table S1. List of antibodies used in the study.

Antibody	Company	ICC	WB
mouse anti-β actin IgG_1 (#A5441)	Sigma-Aldrich	1:1000	1:20000
mouse anti-β actin IgG_1 (#MCA5775GA)	AbD Serotec (Bio-Rad)	1:100	1:1000
mouse anti-γ actin IgG_1 (#A8481)	Sigma-Aldrich	1:1000	1:20000
mouse anti-γ actin IgG_{2b} (#MCA5776GA)	AbD Serotec (Bio-Rad)	1:100	1:1000
rabbit anti-total actin C11 (#A2066)	Sigma-Aldrich	1:100	1:1000
mouse anti-total actin AC40 (#A3853)	Sigma-Aldrich	1:100	1:1000
rabbit anti-Cofilin (#C8736)	Cell Signaling	1:400	1:2000
rabbit anti-$pCofilin^{3}$ (#C8992)	Sigma-Aldrich	-	1:250
mouse anti-Cortactin (#sc-55579)	Santa Cruz Biotechnology Inc.	1:50	1:200
mouse anti-FHOD1 (#sc-365437)	Santa Cruz Biotechnology Inc.	1:50	1:200
rabbit anti-Myosin IIa (#M8064)	Sigma-Aldrich	1:100	1:1000
rabbit anti-$pMLC^{18/19}$ (#3674)	Cell Signaling	-	1:500
rabbit anti-$pMLC^{19}$ (#3671)	Cell Signaling	1:100	-
rabbit anti VASP (#3132)	Cell Signaling	1:400	1:500
rabbit anti $VASP^{157}$ (#3111)	Cell Signaling	-	1:500
rabbit anti $VASP^{239}$ (#3114)	Cell Signaling	-	1:500
mouse anti-Arp3 IgG_{2a} (#sc-48344)	Santa Cruz Biotechnology Inc.	1:50	1:200
rabbit anti N-WASP (#4848)	Cell Signaling	-	1:200
mouse anti N-WASP IgG_1 (#sc-271484)	Santa Cruz Biotechnology Inc.	1:50	-
rabbit anti WAVE-2 (#3659)	Cell Signaling	1:50	1:1000
rabbit anti-α Parvin (#8190)	Cell Signaling	1:200	1:1000
mouse anti-$pSrc^{530}$ (#sc-166860)	Santa Cruz Biotechnology Inc.	-	1:200
rabbit anti-$pFAK^{397}$ (#8556)	Cell Signaling	-	1:500
mouse anti-αVβ3 integrin (#MAB1976)	Merck-Millipore	1:100	-
mouse anti-β1 integrin (#CBL481)	Merck-Millipore	1:100	-

Table S1. List of antibodies used in the study (contrinued).

Antibody	Company	ICC	WB
HRP-conjugated anti-rabbit (#7074)	Cell Signaling	-	1:4000
HRP-conjugated anti-mouse (#7076)	Cell Signaling	-	1:4000
donkey anti-mouse-Alexa Fluor® 488 (#A21202)	Invitrogen	1:200	-
donkey anti-mouse-Alexa Fluor® 568 (#A10037)	Invitrogen	1:200	-
donkey anti-rabbit-Alexa Fluor® 488 (#A21206)	Invitrogen	1:200	-
donkey anti-rabbit-Alexa Fluor® 568 (#A10042)	Invitrogen	1:200	-
goat anti-mouse IgG_1-Alexa Fluor® 568 (#A21124)	Invitrogen	1:200	-
goat anti-mouse IgG_{2b}-Alexa Fluor® 488 (#A21141)	Invitrogen	1:200	-
goat anti-mouse IgG_{2a}-Alexa Fluor® 488 (#A-21131)	Invitrogen	1:200	-
donkey anti-rabbit-Alexa Fluor® 647 (#A31573)	Invitrogen	1:200	-

Table S2. List of qPCR primers used in the study.

Primer	Sequence	Amplicon size (nt)	Tm (°C)
**qRT-PCR**			
*ACTB*_f<br>*ACTB*_r	5’→tttcttgacaaaacctaacttgcgc<br>5’→attgtgaactttgggggatgctct	175	61
*ACTG1*_f<br>*ACTG1*_r	5’→gcattgccgacaggatgcag<br>5’→atgcagcaaatgctacgcatctg	239	64
*HPRT1*_f<br>*HPRT1*_r	5’→gaccagtcaacaggggacat<br>5’→gcttgcgaccttgaccatct	165	60
**gDNA analysis**			
*ACTB*_f<br>*ACTB*_r	5’→atggatgatgatatcgccg<br>5’→gaagccctgagcacgggcg	239	60
*ACTG1*_f<br>*ACTG1*_r	5’→cattgagcatggcatcgtcac<br>5’→catgctgcatgccagtgtgat	360	63
